# Supplementary material for: Nucleic Acid Polymers with Accelerated Plasma and Tissue Clearance for Chronic Hepatitis B Therapy
Source: Mol Ther Nucleic Acids. 2017 May 4;8:1–12. doi: 10.1016/j.omtn.2017.04.019 (PMC5466589; doi:10.1016/j.omtn.2017.04.019)
Supplement: Document S1. Figures S1–S8 [file mmc1.pdf]

OMTN, Volume *8*

Supplemental Information

Nucleic Acid Polymers with Accelerated Plasma and Tissue Clearance for Chronic Hepatitis B Therapy

Ingo Roehl, Stephan Seiffert, Celia Brikh, Jonathan Quinet, Catherine Jamard, Nadine Dorfler, Jennifer A. Lockridge, Lucyna Cova, and Andrew Vaillant

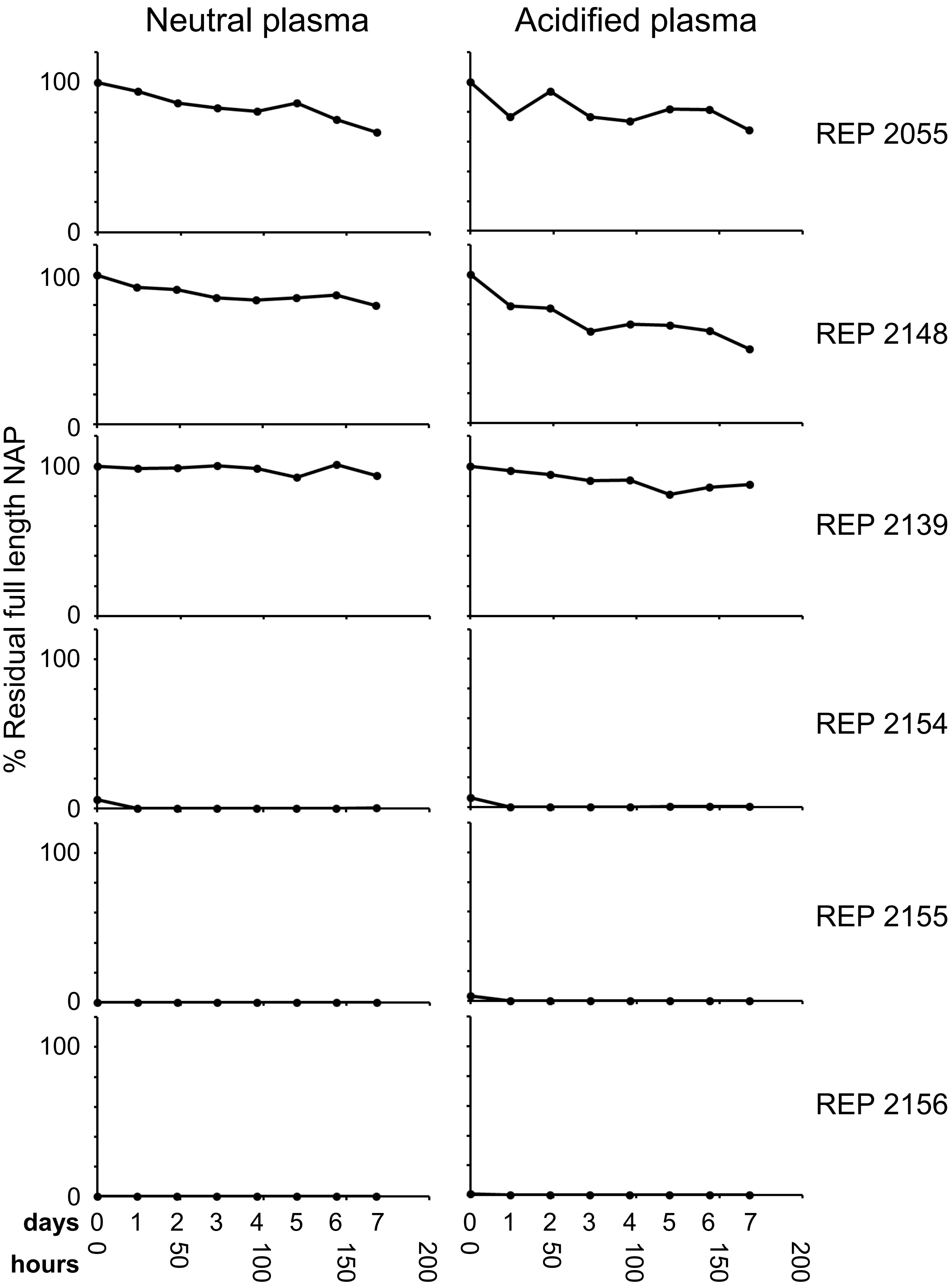

Figure S1. Stability of various NAPs in neutral and acidified human plasma over 7 days (part 1 of 3).

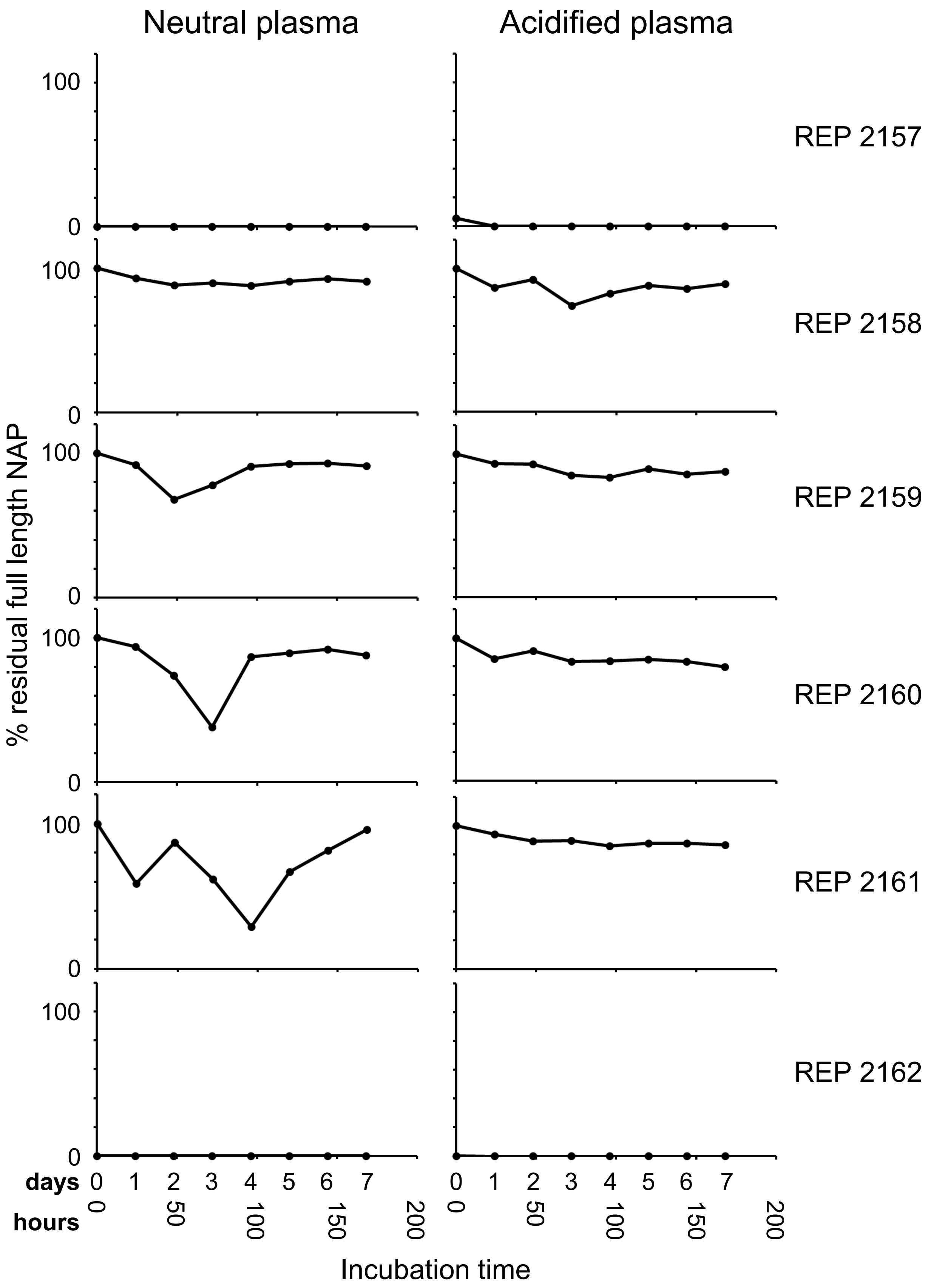

Figure S2. Stability of various NAPs in neutral and acidified human plasma over 7 days (part 2 of 3).

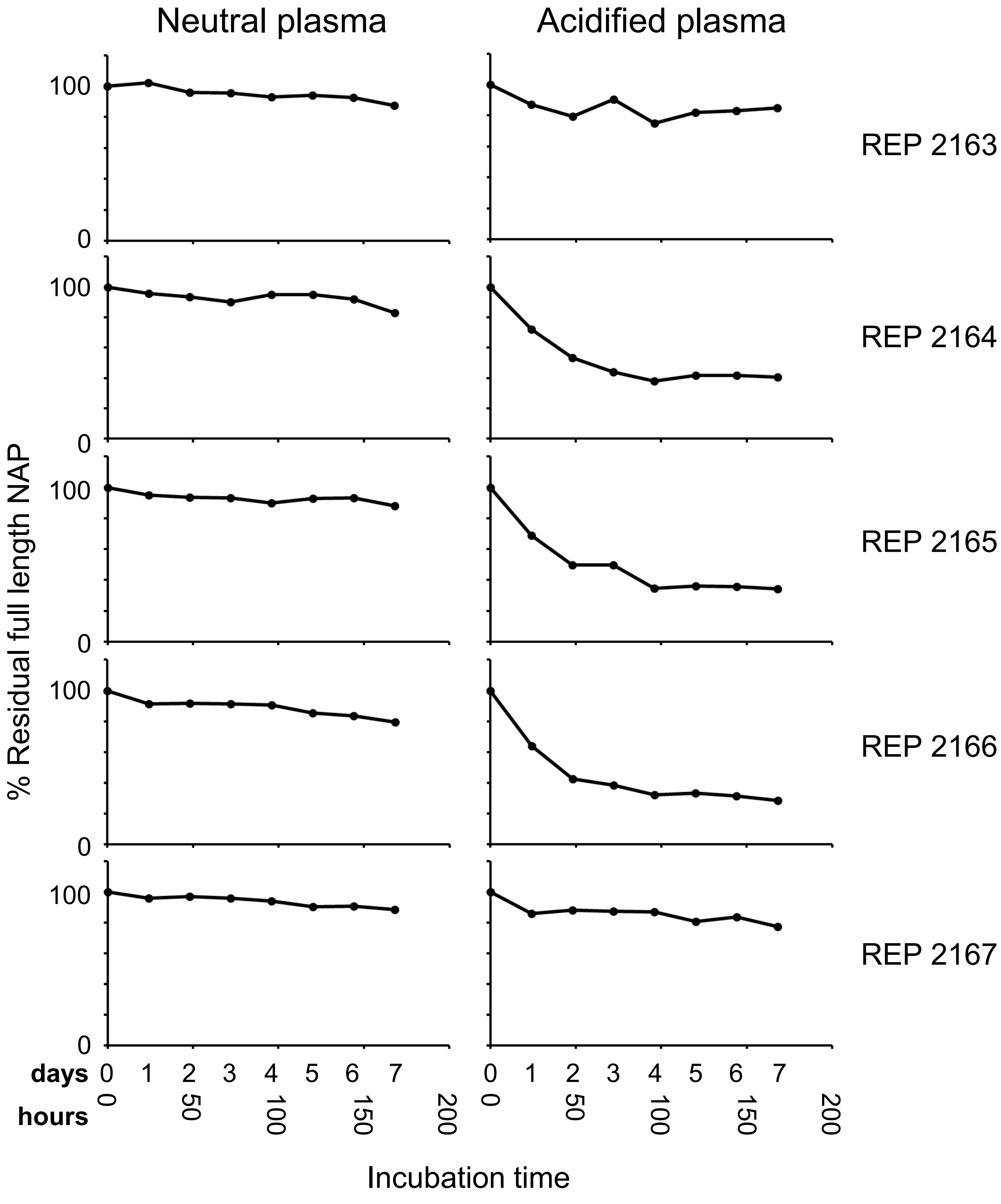

Figure S3. Stability of various NAPs in neutral and acidified human plasma over 7 days (part 3 of 3).

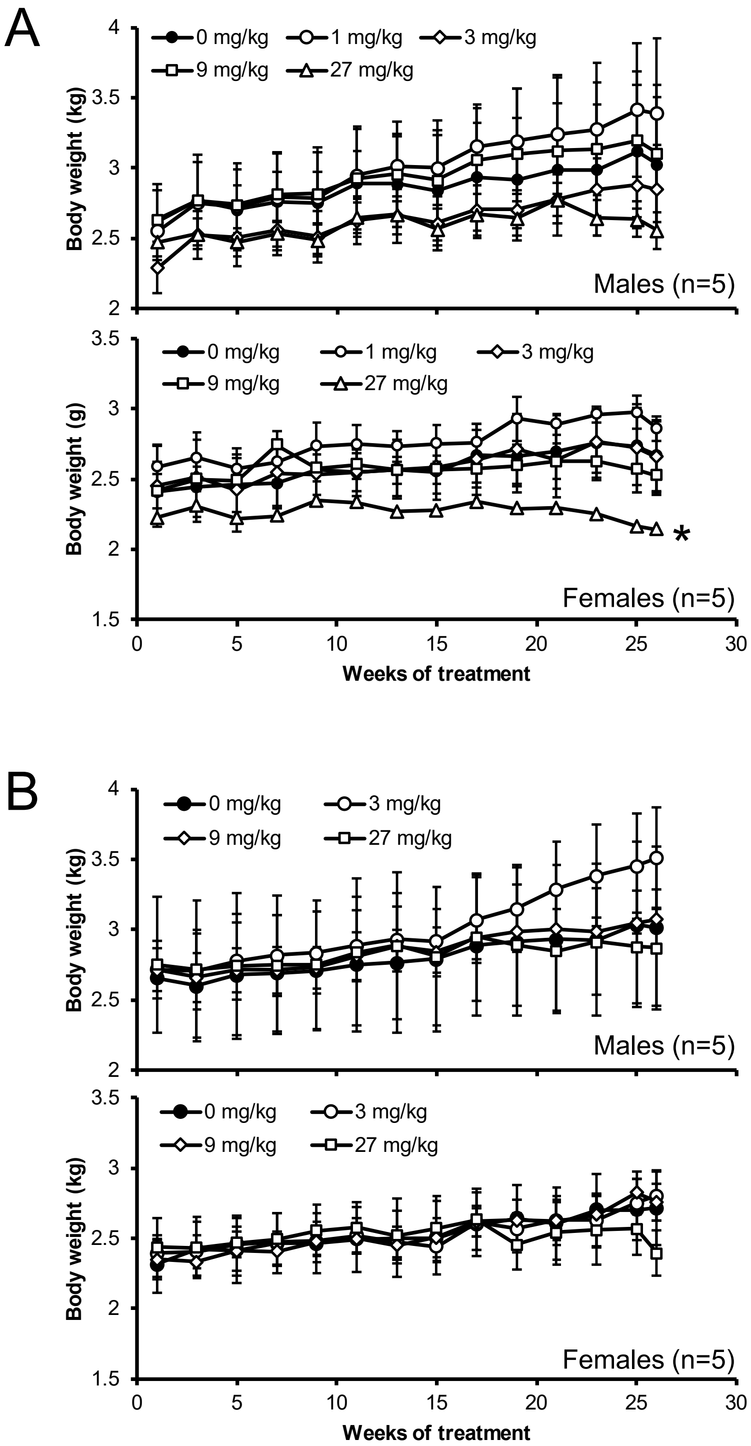

Figure S4. Body weight changes in cynomolgus monkeys over 26 weeks in the presence of various doses of REP 2139 (A) and REP 2165 (B) administered weekly by intravenous infusion. Mean and standard deviation are plotted separately for and females. Weight trends significantly different ($p < 0.05$, as determined by one-way ANOVA) from the 0 mg/kg control are indicated with an asterisk.

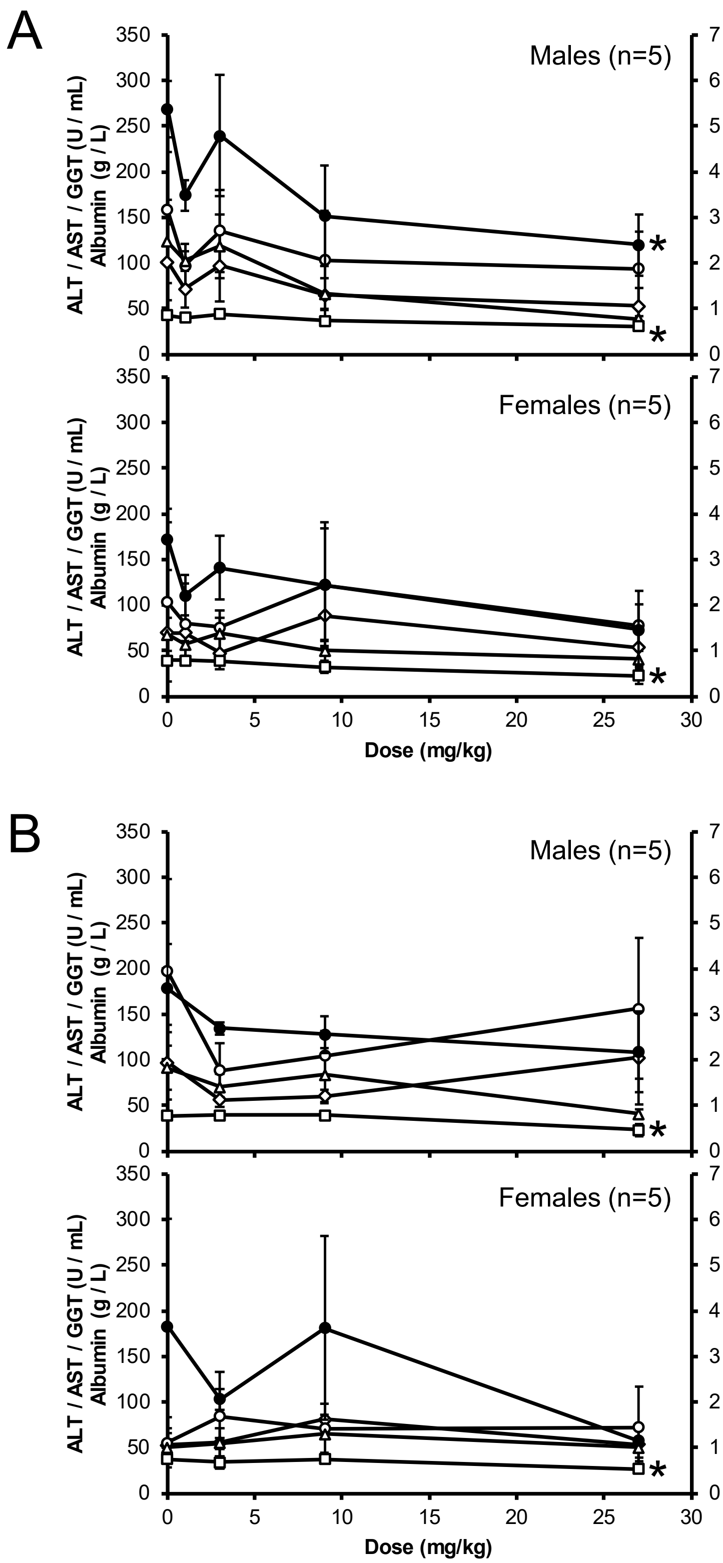

Figure S5. Liver function changes in cynomolgus monkeys at the end of 26 weeks of various doses of REP 2139 (A) and REP 2165 (B) administered weekly by intravenous infusion. Mean and standard deviation of ALT (diamonds), AST (open circles), GGT (triangles), albumin (squares) and total bilirubin (closed circles) are plotted separately for males and females. Trends significantly different ($p < 0.05$, as determined by one-way ANOVA) from the 0 mg/kg control are indicated with an asterisk.

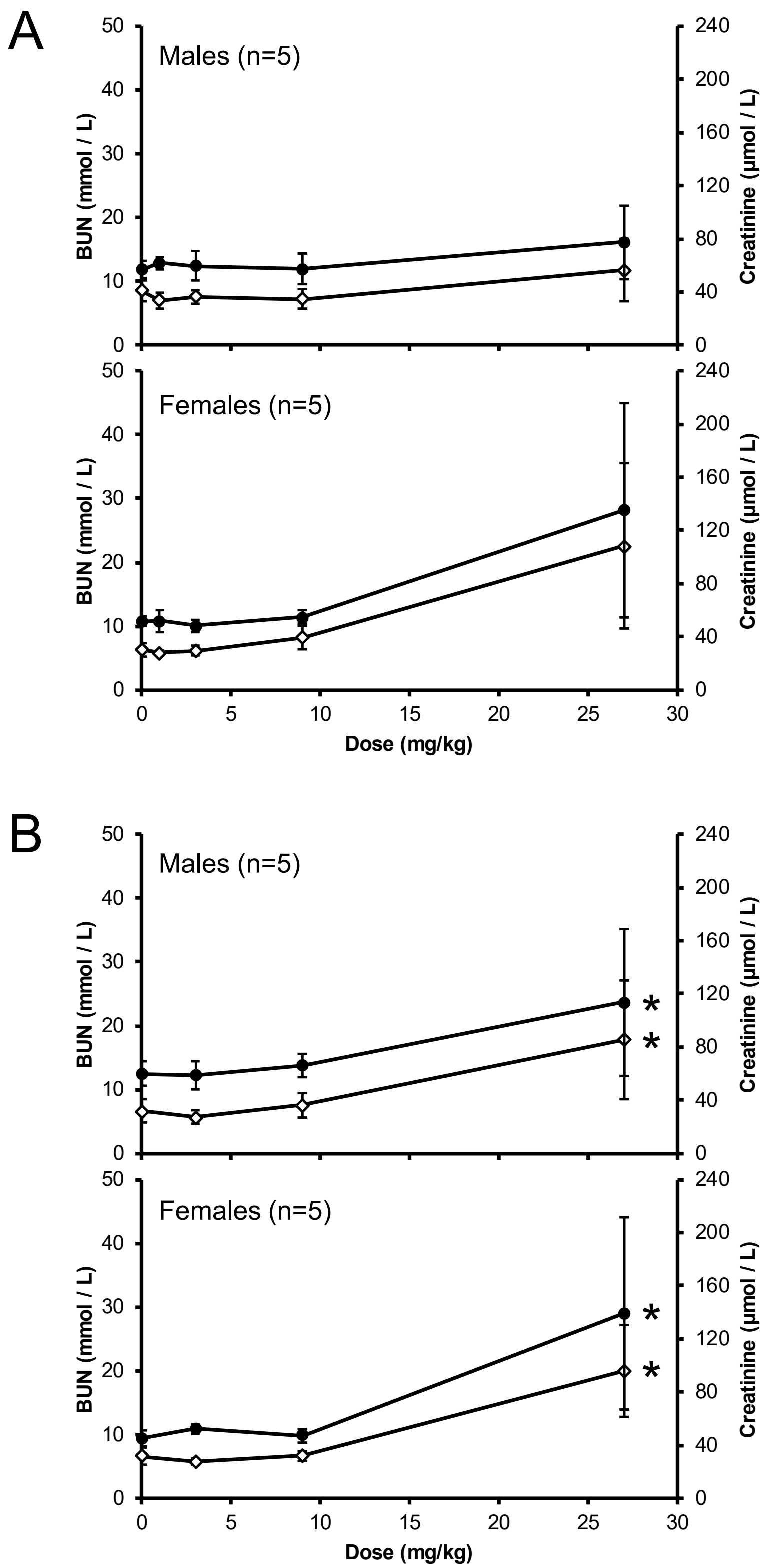

Figure S6. Kidney function changes in cynomolgus monkeys at the end of 26 weeks of various doses of REP 2139 (A) and REP 2165 (B) administered weekly by intravenous infusion. Mean and standard deviation of BUN (diamonds) and creatinine (closed circles) are plotted separately for males and females. Trends significantly different ($p < 0.05$, as determined by one-way ANOVA) from the 0 mg/kg control are indicated with an asterisk. BUN and creatinine increases in females at 27 mg/kg (whether significant or not) were considered clinically relevant.

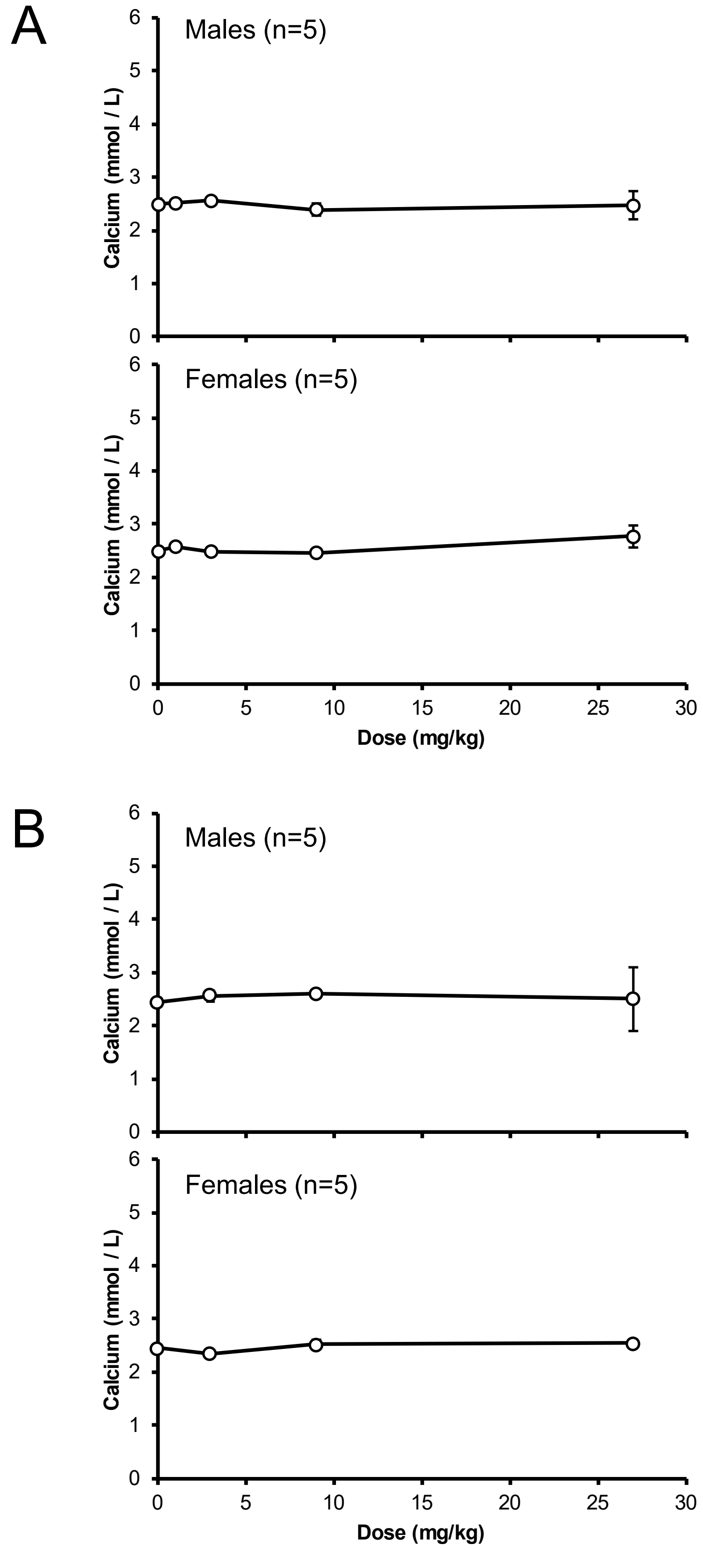

Figure S7. Serum calcium changes in cynomolgus monkeys at the end of 26 weeks of various doses of REP 2139 (A) and REP 2165 (B) administered weekly by intravenous infusion. Mean and standard deviation are plotted separately for males and females.

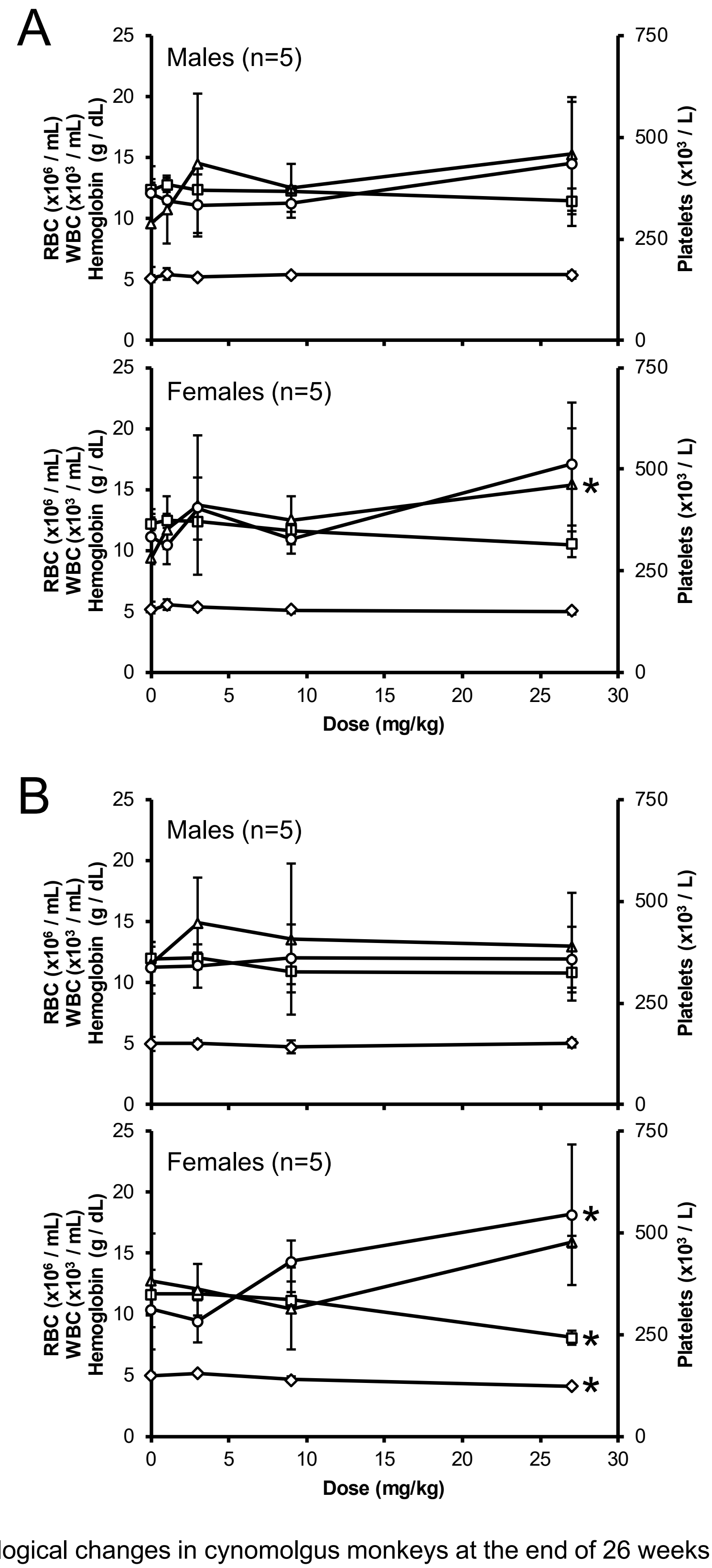

Figure S8. Hematological changes in cynomolgus monkeys at the end of 26 weeks of various doses of REP 2139 (A) and REP 2165 (B) administered weekly by intravenous infusion. Mean and standard deviation of RBC (diamonds), WBC (triangles), hemoglobin (squares), and platelets (circles) are plotted separately for males and females. Trends significantly different ($p < 0.05$, as determined by one-way ANOVA) from the 0 mg/kg control are indicated with an asterisk. Alterations in RBC and platelets at the 27mg/kg dose were not considered adverse.
