# Supplementary material for: Nucleic Acid Polymers with Accelerated Plasma and Tissue Clearance for Chronic Hepatitis B Therapy
Source: Mol Ther Nucleic Acids. 2017 May 4;8:1–12. doi: 10.1016/j.omtn.2017.04.019 (PMC5466589; doi:10.1016/j.omtn.2017.04.019)
Supplement: Document S2. Article plus Supplemental Information [file mmc2.pdf]

Molecular Therapy
Nucleic Acids
Original Article

AMERICAN SOCIETY of GENE & CELL THERAPY

# Nucleic Acid Polymers with Accelerated Plasma and Tissue Clearance for Chronic Hepatitis B Therapy

Ingo Roehl,[1,5] Stephan Seiffert,[1] Celia Brikh,[2] Jonathan Quinet,[2] Catherine Jamard,[2] Nadine Dorfler,[1] Jennifer A. Lockridge,[3] Lucyna Cova,[2] and Andrew Vaillant[4,5]

[1]Axolabs, GmbH, Kulmbach 95326, Germany; [2]Inserm U0152, Lyon 69003, France; [3]Lockridge Pharmaceutical Consulting, LLC, Westminster, CO 80021, USA; [4]Replicor, Inc., Montreal, QC H4P 2R2, Canada

**REP 2139 is a nucleic acid polymer (NAP) currently under clinical development for chronic hepatitis B (HBV) therapy. This preclinical study investigated different REP 2139 analogs that would display reduced accumulation in the serum and tissues, while retaining an antiviral effect against HBV infection. REP 2139 analogs were evaluated in human plasma, CD-1 mice, cynomolgus monkeys, and Pekin ducks. Discrete ribose transformation to 2′OH in selected riboadenosines resulted in a slow degradation in acidified human plasma that plateaued after 48 hr. REP 2165, a REP 2139 analog containing three unmodified riboadenosines equally spaced throughout the polymer, showed similar plasma clearance and tissue distribution as REP 2139 in mice and cynomolgus monkeys after a single dose. Interestingly, after repeated administration, accumulation of REP 2165 in plasma and organs was reduced, indicating a dramatically faster rate of clearance from organs after therapy was ended in both species. Both REP 2139 and REP 2165 were well tolerated at clinically relevant doses, with no alterations in liver, kidney, or hematological function. In chronic duck HBV (DHBV) infection, REP 2165 displayed significantly reduced liver accumulation after repeated dosing but retained antiviral activity similar to REP 2139. These results indicate the therapeutic potential of REP 2165 against chronic HBV infection in patients is similar to REP 2139, but with significantly reduced drug accumulation and improved tissue clearance.**

## INTRODUCTION

Nucleic acid polymers (NAPs) are oligonucleotide-based, broad-spectrum antiviral agents. Their activity is driven by interactions with large amphipathic protein domains important for viral replication and relies only on the length of the oligonucleotide (optimally 40-mer) and the presence of phosphorothioation.[1] The NAP REP 2139 is currently in clinical development for the treatment of chronic hepatitis B (HBV) infection and HBV/hepatitis delta (HDV) co-infection and has shown a unique ability to clear the HBV surface antigen (HBsAg) from the blood in clinical trials.[2–4] This activity is driven by the ability of NAPs to block the release of HBsAg from infected hepatocytes, likely by interfering with the assembly of HBV subviral particles[5] by an as yet undefined mechanism. Importantly, the clearance of HBsAg from the circulation by the NAPs REP 2055 and REP 2139 is associated with a drastic clearance of intrahepatic hepadnaviral infection from the liver (HBsAg, HBcAg, total and covalently closed circular DNA [cccDNA]) that persists after NAP therapy cessation, indicating a functional control of infection has been established.[5,6] The elimination of serum HBsAg also improves the efficacy of immunotherapy in human patients,[2–4] which may facilitate the achievement of a functional cure (restoration of host immune control of HBV infection) in the absence of treatment.

The optimization of NAPs for clinical use has been previously described[1] and has resulted in the design of an NAP with an optimal length (40-mer), fully phosphorothioated, having a repetitive sequence (adenosine-cytidine [AC]), and incorporating 5-methyation of all cytosines and 2′O-methyl modification of all riboses (REP 2139). These optimizations preserve antiviral activity against HBV while preventing recognition by the innate immune response[7–12] to allow their safe use with immunotherapies such as pegylated interferon. One of the class effects of administration of phosphorothioate oligonucleotides (PS-ONs) is an increased elimination of divalent metal minerals in the urine such as magnesium, calcium, and iron, an effect attributed to the chelation of these divalent metals by non-bridging oxygen or sulfur atoms in the phosphodiester linkage.[13] Symptoms consistent with mineral deficiency have not been reported in previous clinical trials with PS-ONs; however, PS-ON-mediated mineral elimination is likely well tolerated in patients with adequate dietary access to these minerals and with normal endocrine function to compensate for increased mineral loss by bone resorption. Both REP 2139 and its clinical progenitor, REP 2055, have the same pharmacologic effect, but REP 2055 is more labile to nuclease-mediated

Received 19 January 2017; accepted 28 April 2017;
http://dx.doi.org/10.1016/j.omtn.2017.04.019.

[5]These authors contributed equally to this work.

**Correspondence:** Andrew Vaillant, Replicor, Inc., 6100 Royalmount Avenue, Montreal, QC H4P 2R2, Canada.
**E-mail:** availlant@replicor.com

CrossMark


This is an open access article under the CC BY license (http://creativecommons.org/licenses/by/4.0/).

**Table 1. NAPs Used in This Study and Their Stability in Human Plasma**

| NAP | Sequence 5′-3′ | 5-MeC[a] | Stability in Human Plasma (7 Days at 37°C) (% of Untreated Standard) Neutral | Acidified |
|---|---|---|---|---|
| REP 2055[b] | ACACACACACACACACACACACACACACACACACACACAC | – | 84 | 69 |
| REP 2148 | ACACACACACACACACACACACACACACACACACACACAC | + | 80 | 53 |
| REP 2139[b] | ACACACACACACACACACACACACACACACACACACACAC | + | 93 | 86 |
| REP 2154 | ACACACACACACacACACACACACACACacACACACACACAC | + | TND | 1 |
| REP 2155 | ACACACACACacacACACACACACACacacACACACACAC | + | TND | TND |
| REP 2156 | ACACACACACacacacACACACACacacacACACACACAC | + | TND | TND |
| REP 2157 | ACACACACACacACACACACacACACACACacACACACAC | + | TND | TND |
| REP 2158 | ACACACACACACACACACACACACACACACACACACACAC | + | 90 | 92 |
| REP 2159 | ACACACACACACACACACACACACACACACACACACACAC | + | 92 | 84 |
| REP 2160 | ACACACACACACACACACACACACACACACACACACACAC | + | 91 | 83 |
| REP 2161 | ACACACACACACACACACACACACACACACACACACACAC | + | 89 | 83 |
| REP 2162 | AcAcAcAcAcAcAcAcAcAcAcAcAcAcAcAcAcAcAcAc | + | TND | TND |
| REP 2163 | ACACACACACACACACACACACACACACACACACACACAC | + | 92 | 89 |
| REP 2164 | ACACACACACACaCACACACACACACACaCACACACACACAC | + | 82 | 41 |
| REP 2165 | ACACACACACaCACACACACACaCACACACACaCACACACAC | + | 89 | 36 |
| REP 2166 | aCaCaCaCaCaCaCaCaCaCaCaCaCaCaCaCaCaCaCaC | + | 82 | 28 |
| REP 2167 | ACACACACACACACACACACACACACACACACACACACAC | + | 89 | 82 |

Underlined nucleotides are deoxyribonucleic acid (2′ ribose = H). Lowercase nucleotides are unmodified RNA (2′ ribose = OH). All other nucleotides contain a 2′O-methyl modification. TND, target (full-length NAP) not detected in plasma.
[a]Where indicated, all cytosine bases contain methylation at the 5′ position (5-MeC).
[b]Clinically active against HBV infection in patients with chronic HBV infection.[2]

degradation due to the absence of 2′O-methylation. The first two proof-of-concept clinical trials with the NAPs REP 2055 (the REP 2139 progenitor; see Table 1) and REP 2139 were the first reported trials with PS-ONs to be conducted in a locale with high endemic exposure to heavy metals (Bangladesh). Retroactive analysis of patients in these trials revealed significant heavy metal loads were present.[2] Although both REP 2055 and REP 2139 had comparable activity against HBV infection, chronic REP 2139 dosing was associated with symptoms consistent with heavy metal intoxication (dysphasia, dysgeusia, and hair loss), which was absent with chronic dosing with REP 2055.[2] This was attributed to an enhanced mineral elimination with REP 2139, likely driven by progressively increased plasma trough concentrations and organ accumulation compared with the more labile REP 2055. The symptoms consistent with heavy metal intoxication occurring with REP 2139 in these patients are likely caused by liberation of heavy metals from the bones during the compensatory response to enhanced mineral elimination. These symptoms have been absent with chronic REP 2139 administration in recent clinical trials where patients had negligible heavy metal exposure.[3,4] Despite the obvious clinical benefits of REP 2139 therapy, the potential need to exclude patients with heavy metal exposure, as well as the likely significant mineral elimination, which could also affect immune function, suggests additional optimization of REP 2139 to reduce its systemic exposure with chronic dosing would be beneficial for patients in locales where heavy metal exposure is pervasive.

The purpose of this study was to design an NAP with increased lability (like REP 2055) without sacrificing the improved solubility and lack of recognition by the innate immune response of REP 2139. Optimally, increased degradation would be desired only intracellularly so as not to prevent uptake of full-length NAP in target cells in the liver. A series of NAPs derived from the structure of REP 2139 were prepared where the 2′O-methyl modifications present were modified in selected nucleotides to 2′OH (unmodified RNA) or 2′H (DNA) to investigate the stability of these NAPs in human plasma. Selected NAP candidates showing the desired degradation properties were subjected to pharmacokinetic and tissue distribution analysis in CD-1 mice, cynomolgus monkeys, and Pekin ducks. In addition, the antiviral effects of these selected NAPs were evaluated in Pekin ducks chronically infected with duck HBV (DHBV), a preclinical model of HBV infection shown to reliably predict the antiviral effects of REP 2055 and REP 2139 in human patients.[2,3,5,6,14] This work resulted in the identification of REP 2165, a novel REP 2139 analog exhibiting an improved pharmacokinetic profile that retained antiviral activity in the DHBV model of infection.

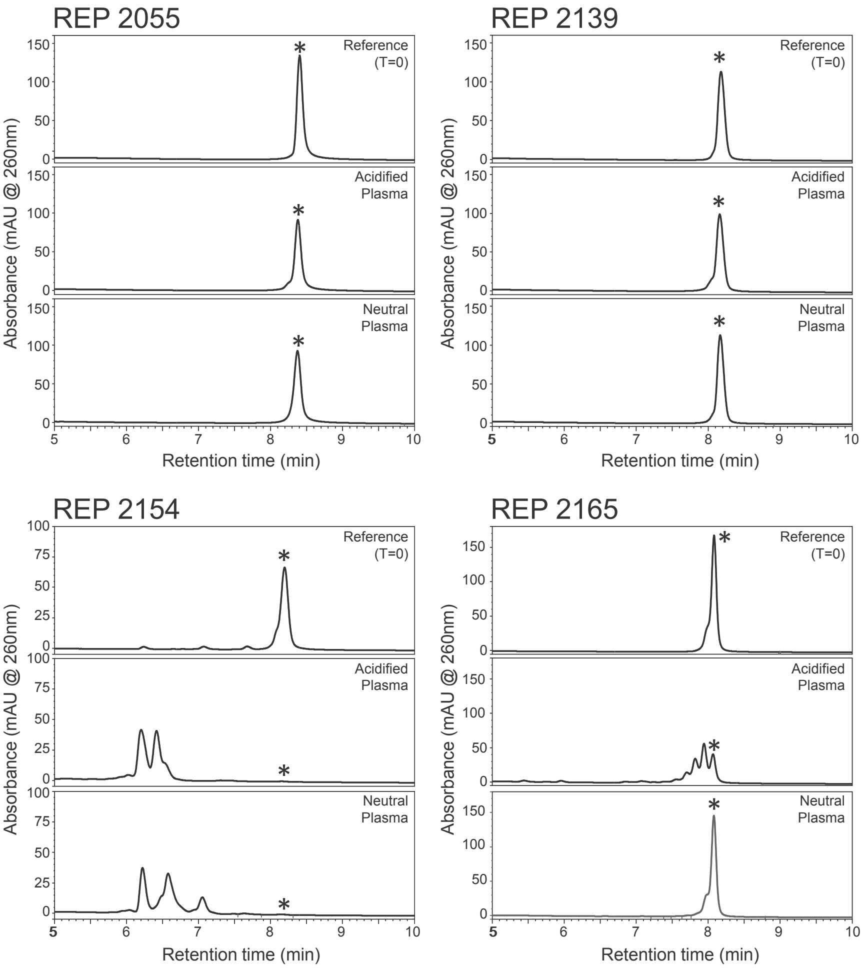


**Figure 1. FL-HPLC Analysis of Degradation of Selected NAPs in Neutral or Acidified Human Plasma after 7 Days of Incubation at 37°C**
Asterisks (*) indicate parental 40-mer NAP species.

## RESULTS

### Stability of Various REP 2139 Derivatives in Human Plasma

Degradation of oligonucleotides in vivo occurs primarily via exonuclease (endwise)-mediated and endonuclease (internal)-mediated cleavage. Degradation via exonucleases can occur in the plasma and intracellularly, whereas endonuclease activity requires acidic pH and occurs mostly intracellularly.[15–20] The endonuclease RNase A is an exception to this and is also active at neutral pH in plasma. For REP 2139, resistance to these modes of degradation is provided by phosphorothioation and 2′O-methylation of ribose nucleotides, respectively.[21] Because phosphorothioation is the key driver of antiviral activity of NAPs,[1] removal of phosphorothioation at selected phosphodiester linkages was not attempted in order to preserve the full pharmacological effect of NAPs and to ensure some measure of stabilization to exonuclease and endonuclease attack was maintained over the entire NAP molecule. Instead, REP 2139 derivatives were prepared where the 2′O-methylation of ribose was replaced by 2′OH (unmodified RNA) or 2′H (DNA) at selected nucleotides (see Table 1). These modifications were designed to allow increased endonuclease attack at specific locations along the NAP in order to accelerate the degradation of the 40-mer parental compound into smaller species more readily cleared.

Because current clinical dosing regimens with NAPs employ administration once each week, NAPs were incubated in human plasma for 7 days at 37°C under neutral (pH 7.4) or acidified (pH 4.5) conditions to simulate intracellular endonuclease activity. The proportion of full-length NAP was assessed every 24 hr relative to un-incubated NAP standard using fluorescence high pressure liquid chromatography (FL-HPLC) (Figures S1–S3). Summarized results of these stability tests are presented in Table 1. REP 2139 was the most stable of all NAPs, and its progenitor REP 2055 showed moderately increased degradation after 7 days. The stability of REP 2055 and its counterpart containing 5′methylcytosine (REP 2148) were comparable. The degradation of REP 2055 was predominantly by exonuclease attack because no products consistent with interior cleavage were detected (Figure 1). Introduction of 2′OH on selected AC dimers (REP 2154, REP 2155, REP 2156, and REP 2157) resulted in an almost instantaneous and complete degradation of NAPs into small fragments (Table 1; Figure 1; Figures S1 and S2). Replacement of RNA by DNA in selected AC dimers (REP 2158, REP 2159, REP 2160, and REP 2161) or on all cytidines (REP 2163) or all adenosines (REP 2167) had little effect on increasing the nuclease sensitivity of NAPs relative to REP 2139 (Table 1; Figures S2 and S3). Introduction of 2′OH on selected adenosines (REP 2164, REP 2165, and REP 2166) resulted in selective degradation in acidified plasma, the bulk of which occurred over the first 48 hr (Table 1; Figure S3). Notably, the degradation products consistent with internal cleavage in REP 2165 were present at day 7 (Figure 1).

### Comparison of Pharmacokinetic Behavior of REP 2139 and REP 2165

Based on its selective degradation in acidified plasma, the plasma-concentration profile of REP 2165 was evaluated in comparison with REP 2139 in CD-1 mice and cynomolgus monkeys, species known to reliably model the pharmacokinetic behavior of PS-ONs in humans.[22,23] The plasma-concentration profile of REP 2139 and REP 2165 at several different doses was comparable in mice and

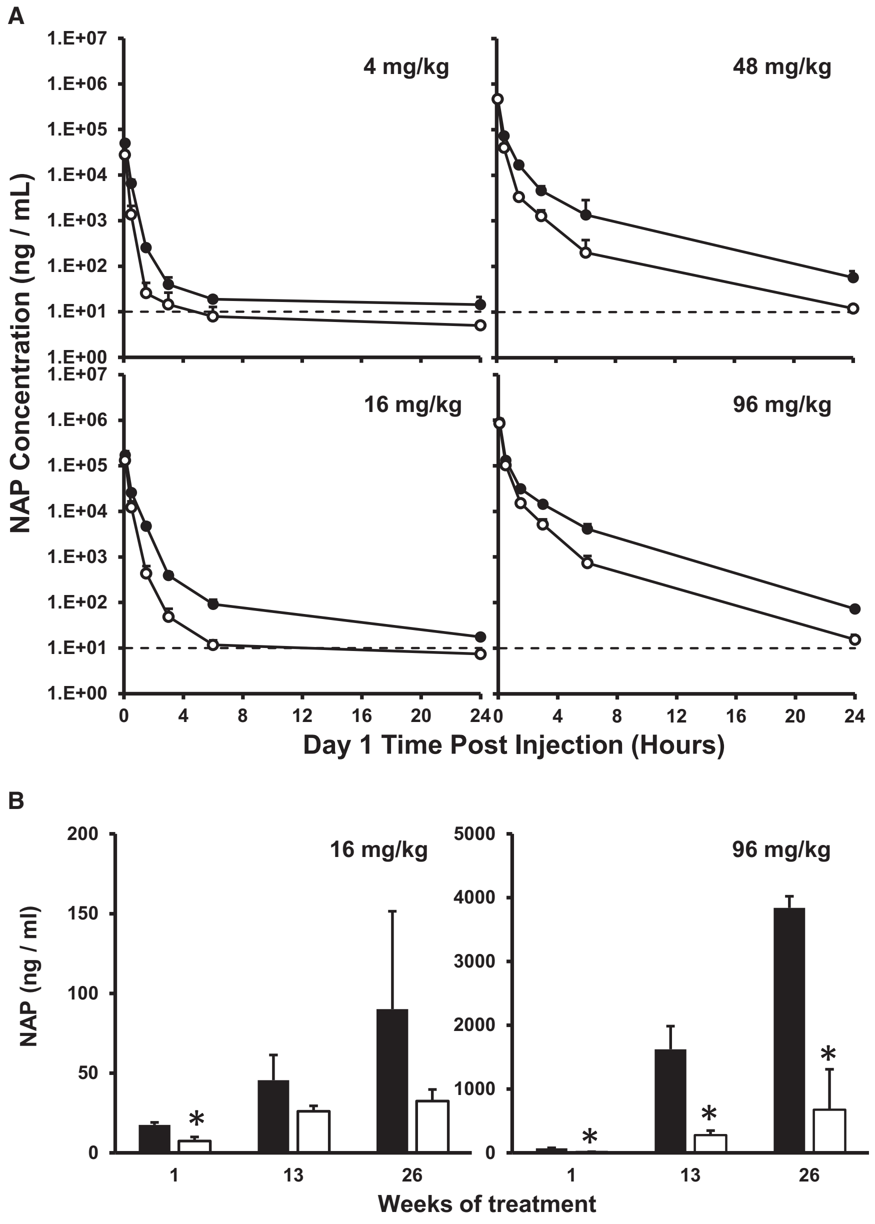


**Figure 2. Plasma Concentrations of REP 2139 and REP 2165 in the CD-1 Mouse**

(A and B) NAP clearance from mouse plasma following the first i.v. bolus injection (A) and NAP concentrations in mouse plasma 24 hr after injection on various days during chronic dosing (B). Dotted lines indicate lower limit of quantification (LLOQ; 10 ng/mL). Values less than the LLOQ (10 ng/mL) were set to 5 ng/mL for calculation of means and SDs. Plotted values = mean ± SD (n = 4 [2 male/2 female]). Asterisks (*) indicate statistically significant difference between REP 2139 (black bars/markers) and REP 2165 (white bars/markers) ($p < 0.05$) as determined by t test.

monkeys, with a biphasic curve occurring over the first 4 hr in mice at all doses evaluated and at the 3 mg/kg dose in monkeys (Figures 2A and 3A). Modest plasma accumulation of REP 2139 was observed with repeated administration, an effect that was much weaker with REP 2165 (Figures 2B and 3B).

Distribution of REP 2139 and REP 2165 in mouse tissues was also similar, with the highest concentrations in the kidney and liver (Figure 4A). However, with repeated dosing, the accumulation of NAPs in mouse and monkey kidney and liver over time was substantially higher with REP 2139 compared with REP 2165 (Figures 4B and 5A). Although the initial dose of either NAP resulted in higher concentrations in kidney versus liver, repeated dosing resulted in greater accumulation in the liver versus the kidney. Importantly, the clearance of REP 2165 from monkey liver and kidney after the end of treatment was much faster with REP 2165 than with REP 2139 (Figure 5B).

### Toxicology of REP 2139 and REP 2165 in Cynomolgus Monkeys

REP 2139 and REP 2165 were generally well tolerated over 6 months of exposure at doses up to 9 mg/kg with no alterations in body weight gain (Figure S4), liver (Figure S5) or kidney (Figure S6) function, calcium homeostasis (Figure S7), or hematological function (Figure S8). At 27 mg/kg, females receiving REP 2139 displayed significantly lower body weight gain (Figure S4). All animals at the 27 mg/kg dose experienced significant decreases in albumin, but no other liver function parameters (Figure S5). Kidney function was also altered with increases in blood urea nitrogen (BUN) and creatinine, which was more pronounced in females. Calcium homeostasis was not altered with either NAP at any dose. Mild declines in red blood cells (RBCs) were observed in females receiving 27 mg/kg REP 2165, and mild increases in platelet counts were observed in females receiving either NAP at 27 mg/kg (Figure S8). Selective, dose-dependent elevations of partial thromboplastin time (PTT), but not prothrombin time (PT), during infusion were observed with both NAPs, which completely resolved within 24 hr (data not shown). Transient PTT elevations are commonly observed with PS-ONs in monkeys and humans[24] with intravenous (i.v.) infusion and are not considered problematic.

With both NAPs, a dose-dependent complement activation (Bb) was observed subsequent to i.v. infusion at the 9 and 27 mg/kg doses, which self-resolved within 24 hr and was not associated with activation of C5a complement (data not shown). Additionally, a

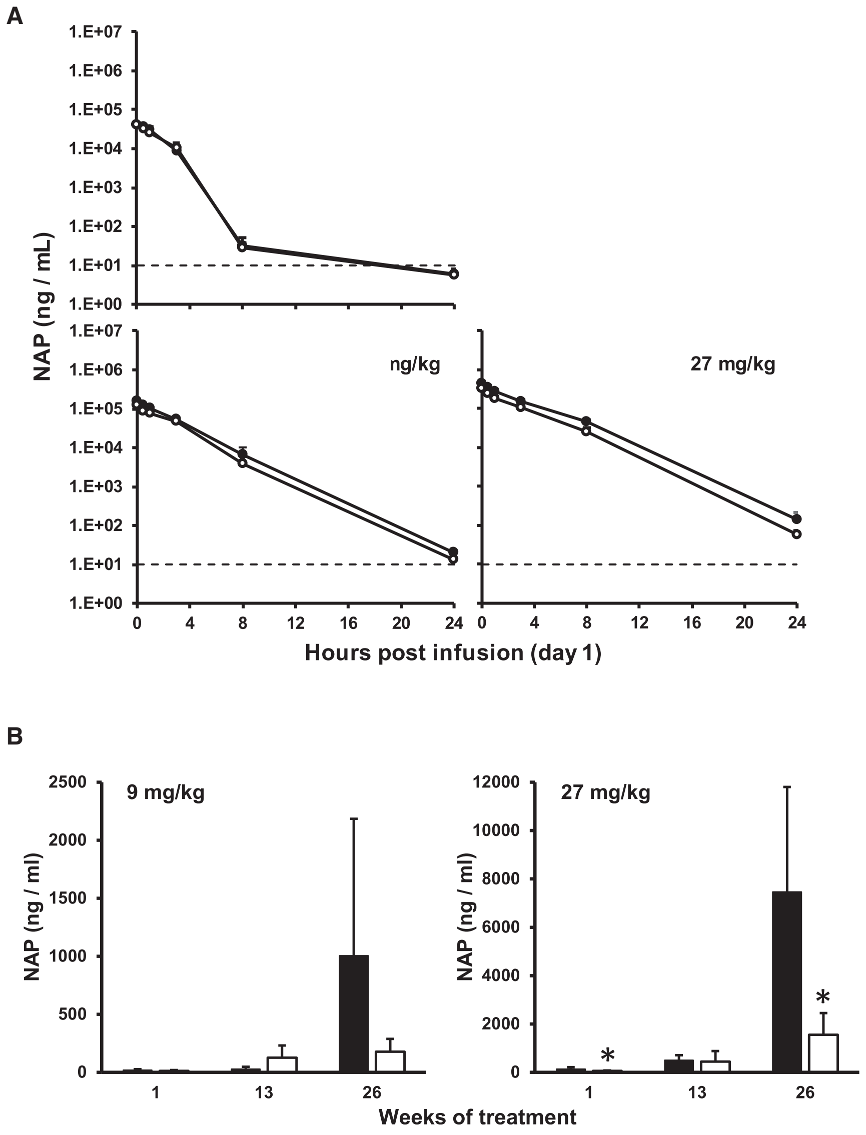


**Figure 3. Plasma Concentrations of REP 2139 and REP 2165 in the Cynomolgus Monkey**

(A and B) NAP clearance kinetics in cynomolgus monkey plasma following the first i.v. bolus injection (A) and NAP concentrations in mouse plasma 24 hr after injection on various days during chronic dosing (B). Dotted lines indicate lower limit of quantification (LLOQ; 10 ng/mL). Values less than the LLOQ (10 ng/mL) were set to 5 ng/mL for calculation of mean and SD. Plotted values = mean ± SD (n = 8–10 [1:1 male:female]). Asterisks (*) indicate statistically significant difference between REP 2139 (black bars/markers) and REP 2165 (white bars/markers) ($p < 0.05$) as determined by t test.

dose-dependent and widely disseminated vasculitis was present at the 9 and 27 mg/kg doses with both NAPs, which appeared more pronounced with REP 2165 (data not shown). This vasculitis was associated with evidence of widespread systemic immune infiltration and altered organ weights, and was the likely underlying cause of altered renal function at the 27 mg/kg dose level. These complications were the likely cause for the early sacrifice of three females (two on day 137 and one on day 165) receiving REP 2165 at 27 mg/kg.

Complement activation is common in cynomolgus monkeys with PS-ONs[25] and is likely the cause of vasculitis, which is common with i.v. PS-ON administration in this monkey species.[26] The complement system of cynomolgus monkeys is highly reactive to PS-ONs, an activity that appears largely absent in humans.[26,27] Additionally, adverse events indicative of complement activation and/or vasculitis are also rarely observed in human patients,[26,27] demonstrating that the observation of complement activation and vasculitis with REP 2139 and REP 2165 in cynomolgus monkeys has little bearing on toxicological effects of these NAPs in human patients.

### Evaluation of NAP Pharmacokinetics and Tissue Biodistribution in the Pekin Duck

The concentration profile of NAPs in Pekin ducks was assessed by examining REP 2139 concentrations in serum, liver, and kidney following a single 9 mg/kg dose given by bolus intraperitoneal (i.p.) injection (Figure 6A). The REP 2139 concentration profile was similar in Pekin ducks compared with mice and monkeys, with rapid clearance from the serum and concomitant accumulation in the liver and kidney. The effect on body weight (Figure 6B) and liver accumulation of various NAPs including REP 2055, REP 2139, REP 2163, REP 2165, and REP 2166 (Figure 6C) was examined in DHBV-infected Pekin ducks following 3 weeks of 10 mg/kg dosage three times per week via i.p. bolus injection. Dosing with all NAPs was well tolerated based on lack of change in body weight. Accumulation of various NAPs was consistent with relative stabilities observed in human plasma; thus, REP 2139 and REP 2163 had the highest concentrations in the liver, whereas REP 2055 and REP 2165 had significantly lower liver concentrations. Administration of REP 2166 resulted in the lowest level of accumulation in the liver.

### Antiviral Effect of Various NAPs in Chronically DHBV-Infected Pekin Ducks

The antiviral effects against DHBV infection in Pekin ducks of various REP 2139 derivatives optimized for enhanced degradation were assessed at the end of 14 days of treatment (Figure 7) in the same ducks that were used to assess liver accumulation of NAPs as described above. Both REP 2055 and REP 2139 were included as control NAP compounds with proven antiviral activity in the duck model[5,6] and in clinical studies.[2,3]

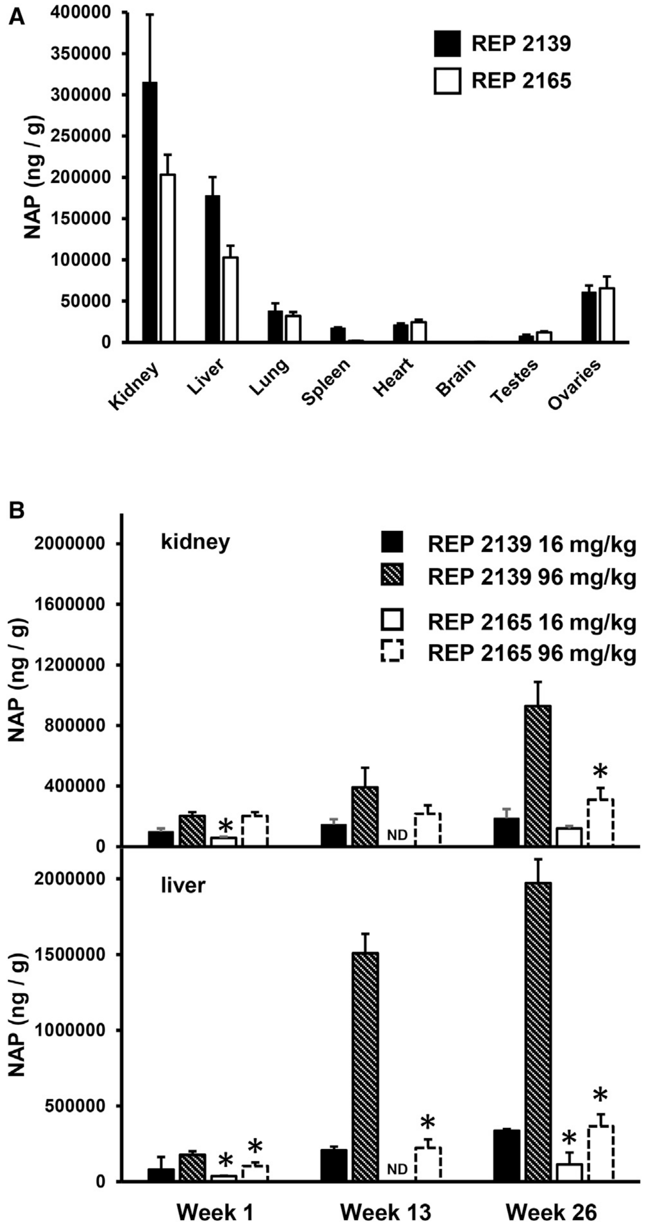


**Figure 4. NAP Tissue Concentrations in CD-1 Mice**

(A) Distribution of NAPs in different tissues 6 hr after the first i.v. bolus injection (96 mg/kg). (B) NAP concentrations in kidney and liver with chronic dosing. Plotted values = mean ± SD (n = 4 [2 male/2 female]). Asterisks (*) indicate statistically significant difference between REP 2139 and REP 2165 ($p < 0.05$) at equivalent dose as determined by t test. ND, not determined.

Only a minimal effect on serum DHBsAg and liver DHBV DNA and cccDNA was observed following treatment with REP 2166. By contrast, a marked reduction in serum DHBsAg accompanied by a significant decrease in total viral DNA in the serum and intrahepatic cccDNA were observed for REP 2055 and REP 2139 as compared with normal saline (NS)-treated controls. Moreover, a reduction in serum DHBsAg was associated with significant decreases in serum and intrahepatic DHBV DNA and intrahepatic viral cccDNA in the REP 2165-treated duck group (Figures 7A–7D).

## DISCUSSION

The goal of this study was to identify REP 2139 analogs that would retain antiviral efficacy against HBV infection in vivo while displaying reduced accumulation in the serum and tissues with repeated dosing. In order to preserve the pharmacological activity of NAPs,[1] phosphorothioation was not altered. The presence of 5′methylcytosine or conversion from 2′OMe-modified RNA to DNA had no effect on nuclease stability. Conversion of selected AC dimers from 2′OMe to 2′OH RNA resulted in rapid and complete cleavage of NAPs, whereas conversion of selected adenosine nucleotides from 2′OMe to 2′OH RNA resulted in a slower and partial cleavage (59%–72%) over 48 hr.

In human plasma a significant RNase A activity is present, an endonuclease that is active at neutral pH. This endonuclease cleaves oligos without 2′ modification at pyrimidines, and rapid degradation of NAPs containing cytidine resides with 2′OH at neutral pH is consistent with this known sensitivity to endonucleases. Interestingly, degradation of NAPs with unmodified riboadenosine nucleotides (REP 2164, REP 2165, and REP 2166) occurred only in acidified plasma and only over the first 48 hr of incubation, leaving a portion of these NAPs resistant to further endonuclease attack over the remaining 5 days of incubation (Figure S3). This persistence was not due to loss of nuclease activity or alteration in pH over time in the plasma (see Materials and Methods), suggesting that some portion of these NAPs was resistant to endonuclease attack. A potential explanation for this behavior is that clockwise-oriented chiral center (Sp) diastereoisomers of phosphorothioated oligonucleotides have been shown to be sensitive to nuclease attack, whereas counter clockwise-orientated chiral center (Rp) diastereoisomers have been shown to be more resistant,[28] and the pools of these NAPs resistant to nuclease attack may represent species with Rp phosphorothioate linkages at the positions of these unmodified riboadenosines.

Another important feature of REP 2139 is its lack of recognition by the pattern recognition sensors of the innate immune response, which improves its tolerability in human patients[2] and does not exacerbate the side effects of immunotherapy when used in combination in patients with chronic HBV infection.[2–4] For this reason, REP 2165 was selected for further evaluation in CD-1 mice and cynomolgus monkeys because it contains only three unmodified riboadenosines, which does not alter the immunoreactivity of this NAP relative to REP 2139.[29] Plasma-concentration profiles of REP 2139 and REP 2165 in mouse and monkey, as well as tissue distribution in the mouse, were similar. However, with repeated dosing, accumulation in plasma and tissue was more pronounced with REP 2139 than REP 2165. Additionally, tissue clearance from monkey liver and kidney after NAP treatment ended was markedly slower with REP 2139 than with REP 2165. REP 2139 has been shown to be well tolerated in patients without heavy metal exposure,[3,4] whereas the more labile REP

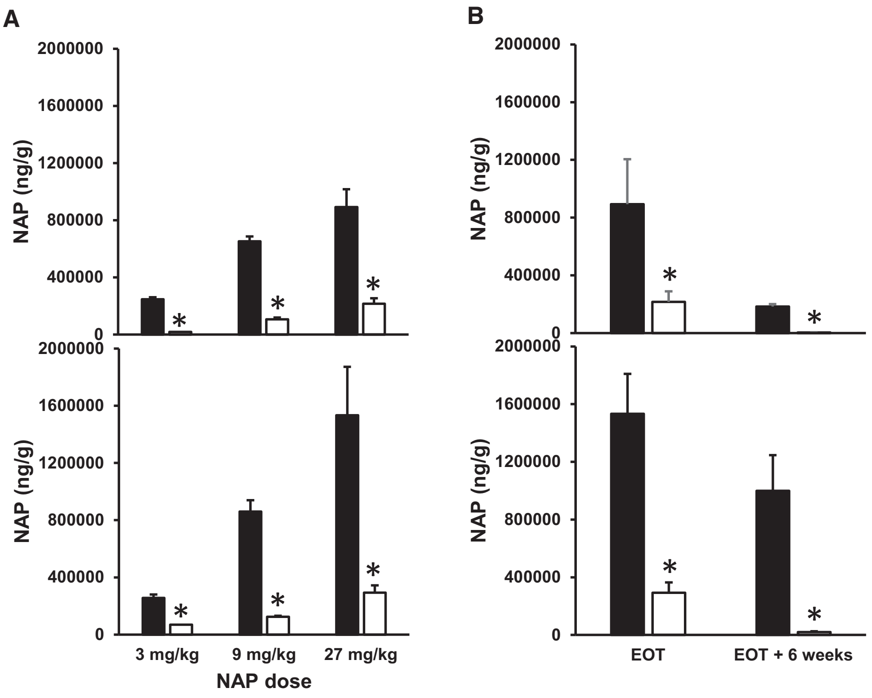


**Figure 5. Tissue Concentrations of REP 2139 and REP 2165 in the Cynomolgus Monkey**

(A) Concentrations of NAPs in monkey kidney (top) and liver (bottom) after 26 weeks of dosing. (B) Concentrations of NAPs 6 weeks after the end of treatment (EOT) following dosing at 27 mg/kg in cynomolgus kidney (top) and liver (bottom). Plotted values = mean ± SD. (A) n = 3–6 (1:1 male:female); (B) n = 4 (2 male, 2 female). Asterisks (*) indicate statistically significant difference between REP 2139 (black bars) and REP 2165 (white bars) ($p < 0.05$) as determined by t test.

2055 was well tolerated in patients with chronic heavy metal exposure,[2] indicating that lowering the level of systemic exposure encountered with chronic dosing is key to allowing NAPs to be used in locales where substantial heavy metal exposure is common. The goal of developing REP 2165 was to have an NAP with the same modifications blocking pro-inflammatory activity as in REP 2139, but having lower organ accumulation and trough plasma levels with chronic exposure. The clearly reduced organ accumulation and trough plasma concentrations of REP 2165 with chronic exposure relative to REP 2139 demonstrated in this study are important performance improvements that could allow REP 2165 to be safely used in patients with heavy metal exposure because the reduced organ accumulation and plasma concentration observed with this NAP in cynomolgus monkeys is expected to translate well to humans.[30] Additionally, REP 2139 and REP 2165 were comparably well tolerated in cynomolgus monkeys with chronic exposure, consistent with the interim results of the REP 401 protocol recently presented.[4] Importantly, no effects on alanine transaminase (ALT) or aspartate transaminase (AST) were observed in monkeys, even at doses substantially higher than those used clinically, further supporting the observations that the transaminase flares observed with NAP therapy in HBV- or HBV/HDV-infected patients are therapeutic in nature and not directly caused by exposure to NAPs.[2–4]

The onset of side effects with REP 2139 in patients with significant heavy metal exposure is not a function of absolute serum calcium levels, but rather a change in calcium dynamics in patients responding to enhanced mineral loss through bone resorption. No changes in serum calcium or magnesium levels were encountered in the patients in the Bangladeshi studies who were experiencing these side effects.[2] In the present study, the calcium levels with REP 2139 and REP 2165 after 6 months of exposure in cynomolgus monkeys showed no changes in serum calcium at doses exceeding those used clinically.

The chronic DHBV infection model represents a pre-clinical reference for the evaluation of antiviral strategies because DHBV has a very similar gene function and replication pathway compared with human HBV, which has an extremely narrow host range. Indeed, most direct-acting antiviral agents currently approved for the treatment of chronic HBV infection in humans (lamivudine, entecavir, and tenofovir) and therapeutic DNA vaccines have been initially tested in this model.[31,32] The clinical effects of NAPs are also accurately modeled in DHBV-infected ducks.[2,3,5,6,14] In this context, the reduced liver accumulation of NAP species containing unmodified riboadenosines was also confirmed in DHBV-infected Pekin ducks and was similar to the clinically active REP 2055 in this model. The antiviral effects of REP 2055 and REP 2139 in DHBV-infected ducks have been previously established.[5,6,14] The primary effect of NAP treatment is to block the release of the viral surface antigen protein (duck hepatitis B surface antigen [DHBsAg]) from infected hepatocytes, likely by blocking the release of subviral particles that constitute the bulk of circulating DHBsAg (similar to HBsAg in human infection). Elimination of serum DHBsAg is accompanied by reduction of DHBV DNA both in the serum and in the liver and also by a decrease in liver cccDNA, which may suggest the reestablishment of host immune control over the infection.[5] Interestingly, REP 2165 retained antiviral activity in this model comparable with REP 2055 and REP 2139, with significant reductions in DHBsAg, serum, and liver DHBV DNA. Additionally, REP 2139 and REP 2165 had the ability to significantly decrease viral cccDNA concentrations in the liver. This finding is of particular interest because the durable suppression of cccDNA, a viral minichromosome, represents a major challenge in the development of novel approaches against chronic HBV infection. Recently, the comparable antiviral effects of REP 2139 and REP 2165 have been confirmed in the ongoing REP 401 clinical trial.[4] By contrast, REP 2166 did not exhibit any meaningful antiviral activity, consistent with the more rapid degradation of REP 2166 compared with REP 2165.

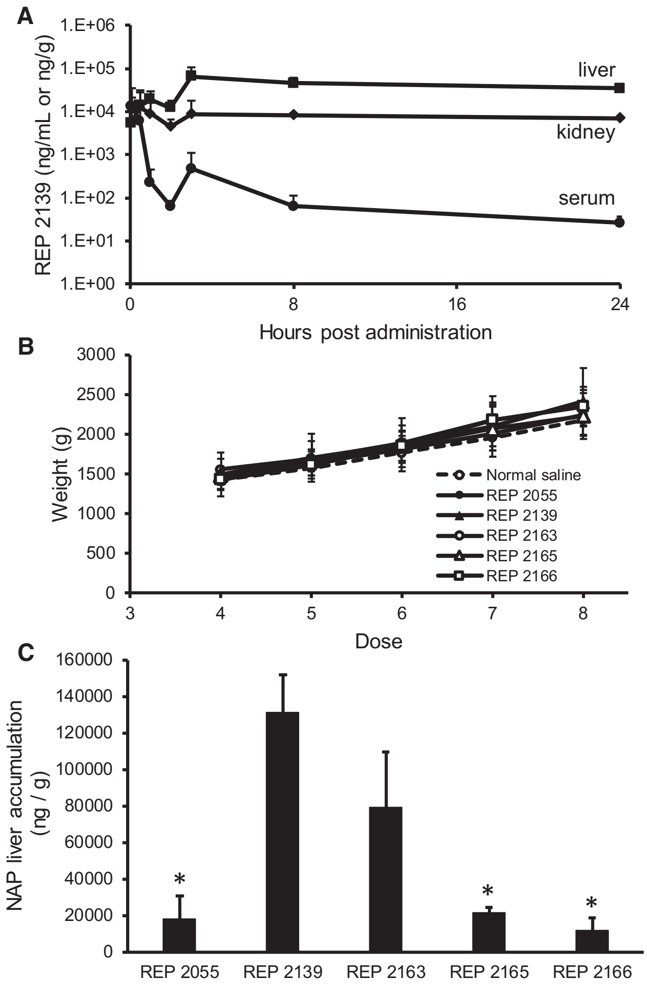


**Figure 6. NAP Serum and Tissue Distribution in the Pekin Duck**

(A) Concentrations of REP 2139 in uninfected duck serum (ng/mL), liver (ng/g), and kidney (ng/g) following a single bolus i.p. administration (9 mg/kg). (B) Body weight in DHBV-infected ducks treated with various NAPs. No statistically significant differences in body weight were observed relative to the saline control group. (C) Liver concentrations of various NAPs after completion of 3 weeks of dosing 10 mg/kg three times per week (see Materials and Methods). All plotted values represent mean ± SD. (A) n = 3; (B and C) n = 5. (C) Asterisks (*) indicate statistically significant difference between liver accumulation of REP 2139 and other NAPs ($p < 0.05$) as determined by t test.

These observations in the duck model, especially with REP 2055, REP 2139, and REP 2165, are important because they demonstrate that pharmacologic activity of NAPs above a certain threshold level of liver accumulation (i.e., between that observed for REP 2165 and REP 2166) is not a function of the extent of accumulation in the liver. Although it is well-known that phosphorothioated oligonucleotides accumulate and are slowly eliminated from the liver, the relationship between this accumulation and pharmacologic activity does not appear to follow the relationship expected from small molecules. In fact, evidence exists for a "non-productive sink" where PS-ONs are shuttled after liver uptake, which does not appear to be related to binding to plasma proteins.[33] This suggests that NAPs (and PS-ONs) may only be active for a short period in the liver following administration, even though they persist for long periods. The comparably active nature of REP 2165 (which is degraded after 2 days) and REP 2139 (which is stable for 7 days) in vivo is consistent with this phenomenon and suggests that the bulk of accumulating NAPs (and PS-ONs) in the liver over the course of chronic therapy represent drug that is sequestered into an as yet unidentified, inactive compartment.

Although the molecular mechanisms underlying the inhibition of HBsAg release by NAPs are not yet elucidated, the results presented herein are consistent with and extend our previous observations demonstrating the post-entry effects of NAPs in vivo in the DHBV infection model.[5,6] Indeed, we reveal here that REP 2139 and REP 2165 are able to significantly decrease the release of surface antigen protein and reduce viral cccDNA in the liver following the treatment of chronic DHBV-carrier animals, thus implying a post-entry effect of NAPs on hepadnaviral replication. The current hypothesis regarding the post-entry NAP mechanism in HBV is that these compounds interfere with a host protein that chaperones the assembly of subviral particles,[1] preventing their assembly and transit through the secretory pathway. Further studies are under way to examine this hypothesis and to get a better insight into the antiviral mechanism of action of REP 2139 and its derivatives.

Collectively, these results suggest that the antiviral activity of REP 2139 observed in patients can be maintained with significantly reduced plasma and tissue accumulation, and that therapy with REP 2165, a novel REP 2139 analog, may allow NAP-based combination therapies to be safely used in patients in any locale. This further suggests that a significant proportion of NAPs accumulating in the liver with chronic dosing are not pharmacologically active, a concept that may translate to other PS-ONs. Importantly, this study provides evidence that plasma and tissue clearance during and after therapy with PS-ONs containing 2′ribose modification can be significantly improved by the use of individual unmodified riboadenosines while retaining pharmacologic activity.

## MATERIALS AND METHODS

### NAP Synthesis

All NAPs used in this study (see Table 1) were prepared via standard solid-phase synthesis routinely used for the preparation of PS-ONs. Purification and conversion to sodium salts were carried out using standard resin-based column purification techniques. Purity and identity of all NAPs were confirmed by high-pressure liquid chromatography-mass spectrometry. NAP compounds for preliminary evaluation of stability in human plasma (Table 1) were synthesized at laboratory scale (mg), and NAP stocks were prepared in normal saline prior to use. Selected NAPs to be tested in Pekin ducks were prepared at small scale (g) and were prepared as calcium chelate complexes in normal saline at 10 mg/mL. For PK/PD studies in CD-1 mice and cynomolgus monkeys, the REP 2139 and REP 2165 active

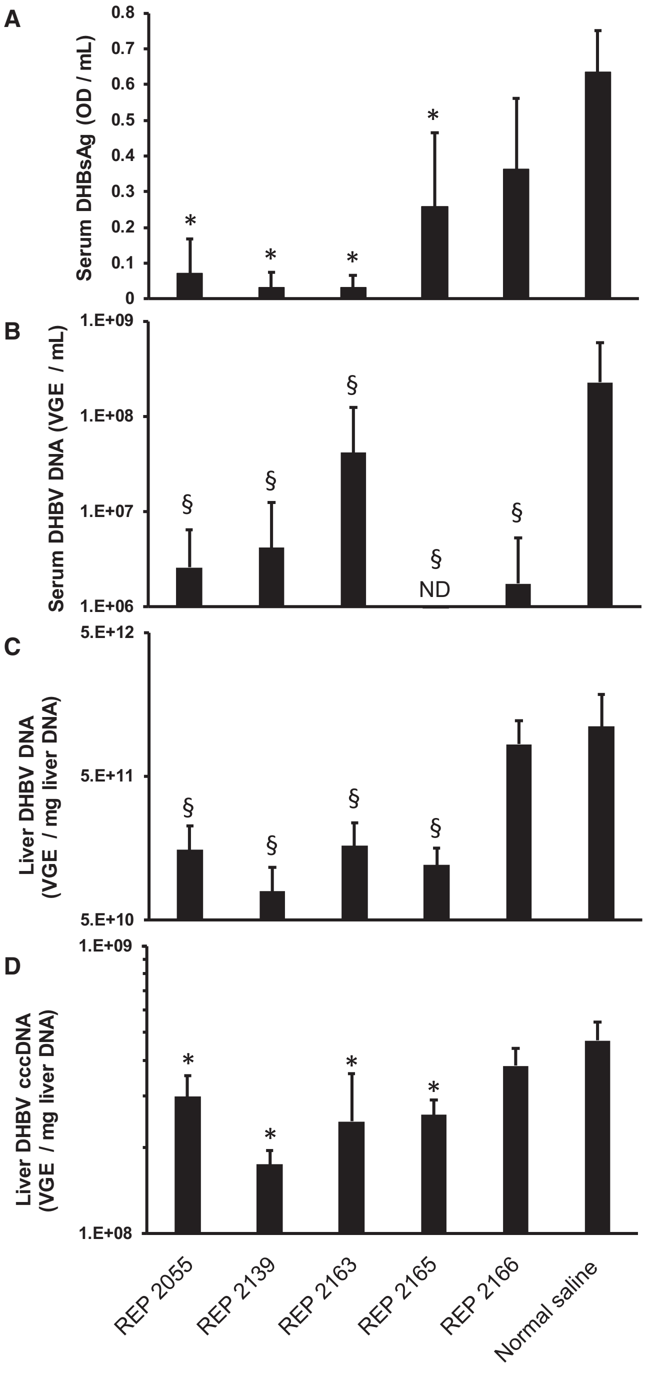


**Figure 7. On-Treatment In Vivo Effects of Various NAPs on Serum and Liver Viremia in DHBV-Infected Pekin Ducks**

(A–D) In vivo effects of various NAPs on (A) serum DHBsAg, (B) serum DHBV DNA, (C) liver DHBV DNA, and (D) liver cccDNA in established DHBV infection in Pekin ducks after 14 days of daily dosing (10 mg/kg i.p. injection). Plotted values = mean ± SD (n = 5). Asterisks (*) indicate statistically significant difference between virologic parameters in normal saline versus NAPs ($p < 0.05$) as determined by t test. Section marks (§) indicate that although not statistically significant, these values represent therapeutically relevant decreases relative to the normal saline group.

pharmaceutical ingredient was prepared under Current Good Manufacturing Practices (cGMP) at midscale (300–400 g). Clinical supply of the drug products REP 2139-Ca (calcium chelate complex, 50 mg/mL in normal saline) or REP 2165-Mg (magnesium chelate complex, 62.5 mg/mL in water) was used to dose mice and monkeys as described below.

### Detection of NAPs in Plasma, Serum, and Tissues

For the detection of NAPs from a biological matrix, we used an HPLC-based assay that is based on the specific hybridization of the NAP sequences with a complementary 28-mer peptide nucleic acid (PNA) probe labeled with an Atto425 fluorescence dye at the N terminus. The PNA probe with the sequence Atto425 5′-OO GTGTGTGTGTGTGTGTGTGTGTGTGTGT-3′ was purchased from Panagene.

Plasma and tissue samples were homogenized by treatment with proteinase K. Snap-frozen tissue samples were grinded on liquid nitrogen; then a weighted aliquot of the tissue powder was treated with proteinase K. Defined aliquots of the plasma samples were also treated with proteinase K before further use. In the resulting proteinase K lysates the NAPs were fully stable because all nucleases present in the biological matrix were degraded.

For the subsequent hybridization step, an aliquot of the proteinase K lysates was mixed with a hybridization buffer containing 3 M urea in 20 mM Tris buffer (pH 8) with 20% acetonitrile and the 28-mer PNA probe. The alternating sequence of NAP sequences allowed multiple hybridizations with the complementary 28-mer PNA probe. Therefore, the hybridization conditions and also the HPLC conditions were tuned to achieve a stoichiometry of one PNA strand per NAP strand. Optimal conditions for the hybridization reaction were achieved by adding 3 M urea into the hybridization buffer. Also, during subsequent HPLC analysis, the increased column temperature of 75°C and the presence of 3 M urea suppressed the formation of non-perfect PNA-NAP duplexes in a 1:2 or 1:3 stoichiometry. The denaturing properties of the urea lead to the selective dissociation of non-perfect PNA-NAP duplexes, whereas mostly perfect duplexes with 28 bp in a 1:1 stoichiometry were formed.

The HPLC analysis was conducted on a Thermo Fisher Scientific Ultimate 3000 HPLC system equipped with a low-pressure gradient pump (1 mL/min), a 96-well plate autosampler, a column compartment at 75°C, and a Shimadzu Florescence detector RF-20Axs (excitation wavelength: 436 nm; emission wavelength: 484 nm). HPLC buffer A was composed of 25 mM Tris-buffer (pH 8), 30% acetonitrile, 1 mM EDTA, and 3 M urea. HPLC buffer B contained 800 mM sodium perchlorate in HPLC buffer A. The HPLC column was washed after each sample using HPLC buffer C with 2.5 M sodium perchlorate in HPLC buffer A. Chromatography was performed on a Thermo Fisher Scientific DNA Pac PA200 column (4 × 250 mm). Gradient elution of the internal standard and the NAP compounds was achieved by increasing the concentration of HPLC buffer B from 8% after 2 min to 55% at 10 min. An aliquot of either

4 μL of plasma or 0.1 mg of tissue was injected per run in 100 μL of the final hybridized sample solution.

The method was fully validated for analysis from plasma, liver, and kidney samples from CD-1 mice and cynomolgus monkey for REP 2139 and REP 2165 (data not shown). For duck plasma and tissue, the method was partially validated for reproducibility, specificity, and linearity in REP 2139. All other NAPs tested have the identical sequence composition, so performance in detecting these NAPs was assumed to be comparable.

### NAP Stability in Neutral and Acidified Human Plasma

The stability of various NAPs was tested in neutral and acidified human plasma. Lyophilized human plasma (anticoagulated with sodium citrate) was purchased from Sigma-Aldrich. The lyophilized human plasma was re-suspended in either water to prepare a neutral human plasma solution or in sodium acetate buffer at pH 4.75 to prepare an acidified human plasma solution. The activation of exonucleases and endonucleases in the neutral and acidified human plasma was confirmed using a nuclease-sensitive oligonucleotide control sequence (Axolabs; data not shown). Persistence of nuclease activity and pH stability over the 7-day incubation was validated by spiking control sequences into plasma at the end of 7 days of incubation at 37°C and observing expected degradation behavior (data not shown).

NAPs were incubated with the neutral or acidified human plasma for up to 7 days at 37°C. At the end of the incubation period the plasma was digested with proteinase K to stop all nuclease-mediated degradation processes. The AEX-HPLC analysis was conducted on a Thermo Fisher Scientific Ultimate 3000 HPLC system equipped with a low pressure gradient (LPG) pump (flow rate: 1 mL/min), 96-well plate autosampler, column oven (75°C), and UV detector. Chromatography was performed on a Thermo Fisher Scientific DNA Pac PA200 column (4 × 250 mm). HPLC buffer A was composed of 25 mM Tris buffer (pH 8), 1 mM EDTA in 50% acetonitrile. HPLC buffer B contained 800 mM sodium perchlorate in HPLC buffer A. Gradient elution of the internal standard and the NAP compounds was achieved by increasing the concentration of HPLC buffer B from 10% after 1 min to 60% at 10 min.

Signals of the internal standard and of the NAP parent compounds together with the related metabolites were recorded at 260 nm. For evaluation of stability, the peak area of the parent compounds at T = 1, 2, 3, 4, 5, 6, and 7 days of incubation in the human plasma were compared with the peak area of the parent compounds at T = 0. The decrease in peak area was calculated to determine stability of the intact parent compound in neutral or acidified human plasma.

### NAP Treatment of CD-1 Mice and Cynomolgus Monkeys

GLP-compliant 6-month toxicity studies in CD-1 mice and cynomolgus monkeys were conducted at Pharmaron. Animals were dosed once weekly for 26 weeks with different doses of REP 2139-Ca or REP 2165-Mg. NAP drug products were diluted in normal saline as required prior to dosing. Administration to mice was performed by a 1- to 2-min bolus i.v. injection in the tail vein. Administration to monkeys was performed by a 2 hr i.v. infusion in normal saline. Harvesting of plasma and tissue samples was done according to standard procedures. All animal procedures were approved by the Pharmaron Institutional Animal Care and Use Committee (IACUC).

### Virus Infection

Pekin ducklings were purchased from a commercial supplier. Chronic DHBV infection was established by inoculation of 3-day-old ducklings with highly infectious duck serum ($2 \times 10^{11}$ DHBV genome equivalents/mL) via i.v. route as previously described.[31] Animal experimentation was performed in accordance with the guidelines of animal care and ethics of the National Veterinary School of Lyon (VetAgro Sup).

### NAP Treatment in Uninfected and DHBV-Infected Pekin Ducks

NAP administration was started in 14-day-old uninfected or chronic DHBV-carrier Pekin ducks and consisted of treatment with 10 mg/kg of different NAPs (REP 2055, REP 2139, REP 2163, REP 2165, and REP 2166), all formulated as calcium chelate complexes in normal saline administered via i.p. injection, three times/week for 3 weeks. The control groups received normal saline administrated by i.p injection following an identical schedule. After the last NAP treatment, all animals were euthanized and the necropsy liver samples were snap-frozen in liquid nitrogen and stored at −80°C until analysis.

### Quantification of Serum and Liver DHBV DNA

DNA was extracted from 100 μL of duck serum using High Pure Viral Nucleic Acids kit (Roche Diagnostics). Total DNA was extracted from frozen necropsy liver samples using NucleoSpin Tissue kit (Macherey-Nagel). DHBV DNA was amplified in a LightCycler 480 (Roche Diagnostics) machine as described previously with minor modifications. In brief, amplification was performed in 1× SYBR Green I Master mix (Roche Diagnostics) containing 20 pmol of each primer 5′-CTGACGGACAACGGGTCTAC-3′ (position 1596–1615) for the forward primer and 5′-GGGTGGCAGAGGAG GAAGT-3′ (position 1728–1746) for the reverse primer. The real-time PCR comprised a denaturation step at 95°C for 1 min 30 s followed by 40 amplification cycles (denaturation at 95°C for 13 s, annealing and elongation at 63°C for 18 s). For quantification of cccDNA, extracted liver DNA (500 ng) was first treated with Plasmid Safe ATP-dependant DNase (Epicenter) to digest the relaxed circular DNA. Thereafter, 4 μL of the digestion product was amplified in 1× SYBR Green I Master mix using previously described cccDNA-specific primers.[34]

### Monitoring of Serum DHBsAg by ELISA

DHBsAg was detected in duck sera using a previously described ELISA test.[14] In brief, microtiter plates (high binding; Greiner) were coated with duck sera, incubated overnight at 37°C, and washed with PBS-0.05% Tween 20. Bound DHBsAg was detected following incubation with anti-DHBV preS monoclonal 1H1 antibody[35] and revealed by horseradish peroxidase-conjugated goat anti-mouse IgG secondary antibody (Life Technologies), followed by visualization

with 3,3′,5,5′-tetramethylbenzidine (TMB) substrate (Life Technologies) and optical density (OD) reading at 450 nm.

## SUPPLEMENTAL INFORMATION

Supplemental Information includes eight figures and can be found with this article online at http://dx.doi.org/10.1016/j.omtn.2017.04.019.

## AUTHOR CONTRIBUTIONS

NAPs were designed by A.V. Experiments were designed by A.V., I.R., and L.C. Experiments were performed by S.S., C.B., J.Q., C.J., and N.D. Data analysis was done by I.R., L.C., J.A.L., and A.V. The paper was written by A.V. with contributions from I.R., L.C., and J.A.L.

## CONFLICTS OF INTEREST

A.V. is an employee and shareholder in Replicor, Inc.

## ACKNOWLEDGMENTS

This work was sponsored by Replicor, Inc. The authors would like to thank Dr. Doug Kornbrust for his invaluable assistance in the design of the NAP studies in mice and monkeys.

## REFERENCES

1. Vaillant, A. (2016). Nucleic acid polymers: broad spectrum antiviral activity, antiviral mechanisms and optimization for the treatment of hepatitis B and hepatitis D infection. Antiviral Res. *133*, 32–40.

2. Al-Mahtab, M., Bazinet, M., and Vaillant, A. (2016). Safety and efficacy of nucleic acid polymers in monotherapy and combined with immunotherapy in treatment-naive Bangladeshi patients with HBeAg+ chronic hepatitis B infection. PLoS ONE *11*, e0156667.

3. Bazinet, M., Pantea, V., Cebotarescu, V., Cojuhari, L., Jimbei, P., Albrecht, J., Schmid, P., Krawczyk, A., Karimzadeh, H., Roggendorf, M., and Vaillant, A. (2016). Update on the safety and efficacy of REP 2139 monotherapy and subsequent combination therapy with pegylated interferon alpha-2a in Caucasian patients with chronic HBV/HDV coinfection. J. Hepatol. *64* (2 Suppl), S584–S585.

4. Bazinet, M., Pantea, V., Placinta, G., Moscalu, I., Cebotarescu, V., Cojuhari, L., Jimbei, P., Larovoi, L., Smesnoi, V., Musteata, T., et al. (2017). Update on safety and efficacy in the REP 401 protocol: REP 2139-Mg or REP 2165-Mg used in combination with tenofovir disoproxil fumarate and pegylated Interferon alpha-2a in treatment naïve caucasian patients with chronic HBeAg negative HBV infection. J Hepatol. *66*, S256.

5. Noordeen, F., Scougall, C.A., Grosse, A., Qiao, Q., Ajilian, B.B., Reaiche-Miller, G., Finnie, J., Werner, M., Broering, R., Schlaak, J.F., et al. (2015). Therapeutic antiviral effect of the nucleic acid polymer REP 2055 against persistent duck hepatitis B virus infection. PLoS ONE *10*, e0140909.

6. Quinet, J., Jamard, C., Vaillant, A., and Cova, L. (2016). Achievement of surface antigen clearance in the liver by combination therapy with REP 2139-Ca and nucleoside analogues against chronic hepatitis B. J. Hepatol. *64* (2 Suppl), S385.

7. Davis, H.L., Weeratna, R., Waldschmidt, T.J., Tygrett, L., Schorr, J., and Krieg, A.M. (1998). CpG DNA is a potent enhancer of specific immunity in mice immunized with recombinant hepatitis B surface antigen. J. Immunol. *160*, 870–876.

8. Karikó, K., Buckstein, M., Ni, H., and Weissman, D. (2005). Suppression of RNA recognition by Toll-like receptors: the impact of nucleoside modification and the evolutionary origin of RNA. Immunity *23*, 165–175.

9. Judge, A.D., Bola, G., Lee, A.C., and MacLachlan, I. (2006). Design of noninflammatory synthetic siRNA mediating potent gene silencing in vivo. Mol. Ther. *13*, 494–505.

10. Robbins, M., Judge, A., Liang, L., McClintock, K., Yaworski, E., and MacLachlan, I. (2007). 2′-O-methyl-modified RNAs act as TLR7 antagonists. Mol. Ther. *15*, 1663–1669.

11. Züst, R., Cervantes-Barragan, L., Habjan, M., Maier, R., Neuman, B.W., Ziebuhr, J., Szretter, K.J., Baker, S.C., Barchet, W., Diamond, M.S., et al. (2011). Ribose 2′-O-methylation provides a molecular signature for the distinction of self and non-self mRNA dependent on the RNA sensor Mda5. Nat. Immunol. *12*, 137–143.

12. Devarkar, S.C., Wang, C., Miller, M.T., Ramanathan, A., Jiang, F., Khan, A.G., Patel, S.S., and Marcotrigiano, J. (2016). Structural basis for m7G recognition and 2′-O-methyl discrimination in capped RNAs by the innate immune receptor RIG-I. Proc. Natl. Acad. Sci. USA *113*, 596–601.

13. Mata, J.E., Bishop, M.R., Tarantolo, S.R., Angel, C.R., Swanson, S.A., and Iversen, P.L. (2000). Evidence of enhanced iron excretion during systemic phosphorothioate oligodeoxynucleotide treatment. J. Toxicol. Clin. Toxicol. *38*, 383–387.

14. Noordeen, F., Vaillant, A., and Jilbert, A.R. (2013). Nucleic acid polymers prevent the establishment of duck hepatitis B virus infection in vivo. Antimicrob. Agents Chemother. *57*, 5299–5306.

15. Tidd, D.M., and Warenius, H.M. (1989). Partial protection of oncogene, anti-sense oligodeoxynucleotides against serum nuclease degradation using terminal methylphosphonate groups. Br. J. Cancer *60*, 343–350.

16. Shaw, J.P., Kent, K., Bird, J., Fishback, J., and Froehler, B. (1991). Modified deoxyoligonucleotides stable to exonuclease degradation in serum. Nucleic Acids Res. *19*, 747–750.

17. Woolf, T.M., Jennings, C.G.B., Rebagliati, M., and Melton, D.A. (1990). The stability, toxicity and effectiveness of unmodified and phosphorothioate antisense oligodeoxynucleotides in Xenopus oocytes and embryos. Nucleic Acids Res. *18*, 1763–1769.

18. Hoke, G.D., Draper, K., Freier, S.M., Gonzalez, C., Driver, V.B., Zounes, M.C., and Ecker, D.J. (1991). Effects of phosphorothioate capping on antisense oligonucleotide stability, hybridization and antiviral efficacy versus herpes simplex virus infection. Nucleic Acids Res. *19*, 5743–5748.

19. Crooke, R.M., Graham, M.J., Cooke, M.E., and Crooke, S.T. (1995). In vitro pharmacokinetics of phosphorothioate antisense oligonucleotides. J. Pharmacol. Exp. Ther. *275*, 462–473.

20. Crooke, S.T., Graham, M.J., Zuckerman, J.E., Brooks, D., Conklin, B.S., Cummins, L.L., Greig, M.J., Guinosso, C.J., Kornbrust, D., Manoharan, M., et al. (1996). Pharmacokinetic properties of several novel oligonucleotide analogs in mice. J. Pharmacol. Exp. Ther. *277*, 923–937.

21. Monia, B.P., Johnston, J.F., Sasmor, H., and Cummins, L.L. (1996). Nuclease resistance and antisense activity of modified oligonucleotides targeted to Ha-ras. J. Biol. Chem. *271*, 14533–14540.

22. Geary, R.S., Leeds, J.M., Henry, S.P., Monteith, D.K., and Levin, A.A. (1997). Antisense oligonucleotide inhibitors for the treatment of cancer: 1. Pharmacokinetic properties of phosphorothioate oligodeoxynucleotides. Anticancer Drug Des. *12*, 383–393.

23. Geary, R.S., Yu, R.Z., Watanabe, T., Henry, S.P., Hardee, G.E., Chappell, A., Matson, J., Sasmor, H., Cummins, L., and Levin, A.A. (2003). Pharmacokinetics of a tumor necrosis factor-alpha phosphorothioate 2′-O-(2-methoxyethyl) modified antisense oligonucleotide: comparison across species. Drug Metab. Dispos. *31*, 1419–1428.

24. Henry, S.P., Monteith, D., and Levin, A.A. (1997). Antisense oligonucleotide inhibitors for the treatment of cancer: 2. Toxicological properties of phosphorothioate oligodeoxynucleotides. Anticancer Drug Des. *12*, 395–408.

25. Shen, L., Frazer-Abel, A., Reynolds, P.R., Giclas, P.C., Chappell, A., Pangburn, M.K., Younis, H., and Henry, S.P. (2014). Mechanistic understanding for the greater sensitivity of monkeys to antisense oligonucleotide-mediated complement activation compared with humans. J. Pharmacol. Exp. Ther. *351*, 709–717.

26. Henry, S.P., Beattie, G., Yeh, G., Chappel, A., Giclas, P., Mortari, A., Jagels, M.A., Kornbrust, D.J., and Levin, A.A. (2002). Complement activation is responsible for acute toxicities in rhesus monkeys treated with a phosphorothioate oligodeoxynucleotide. Int. Immunopharmacol. *2*, 1657–1666.

27. Henry, S.P., Jagels, M.A., Hugli, T.E., Manalili, S., Geary, R.S., Giclas, P.C., and Levin, A.A. (2014). Mechanism of alternative complement pathway dysregulation by a phosphorothioate oligonucleotide in monkey and human serum. Nucleic Acid Ther. *24*, 326–335.

28. Spitzer, S., and Eckstein, F. (1988). Inhibition of deoxyribonucleases by phosphorothioate groups in oligodeoxyribonucleotides. Nucleic Acids Res. *16*, 11691–11704.

29. Real, C.I., Werner, M., Paul, A., Gerken, G., Schlaak, J.F., Vaillant, A., and Broering, R. (2017). Nucleic acid-based polymers effective against hepatitis B Virus infection in patients don't harbor immunostimulatory properties in primary isolated liver cells. Sci. Rep. *7*, 43838.

30. Yu, R.Z., Grundy, J.S., Henry, S.P., Kim, T.W., Norris, D.A., Burkey, J., Wang, Y., Vick, A., and Geary, R.S. (2015). Predictive dose-based estimation of systemic exposure multiples in mouse and monkey relative to human for antisense oligonucleotides with 2′-o-(2-methoxyethyl) modifications. Mol. Ther. Nucleic Acids *4*, e218.

31. Cova, L. (2017). Present and future DNA vaccines for chronic hepatitis B treatment. Expert Opin. Biol. Ther. *17*, 185–195.

32. Cova, L., and Zoulim, F. (2004). Duck hepatitis B virus model in the study of hepatitis B virus. Methods Mol. Med. *96*, 261–268.

33. Geary, R.S., Wancewicz, E., Matson, J., Pearce, M., Siwkowski, A., Swayze, E., and Bennett, F. (2009). Effect of dose and plasma concentration on liver uptake and pharmacologic activity of a 2′-methoxyethyl modified chimeric antisense oligonucleotide targeting PTEN. Biochem. Pharmacol. *78*, 284–291.

34. Khawaja, G., Buronfosse, T., Jamard, C., Abdul, F., Guerret, S., Zoulim, F., Luxembourg, A., Hannaman, D., Evans, C.F., Hartmann, D., and Cova, L. (2012). In vivo electroporation improves therapeutic potency of a DNA vaccine targeting hepadnaviral proteins. Virology *433*, 192–202.

35. Pugh, J.C., Di, Q., Mason, W.S., and Simmons, H. (1995). Susceptibility to duck hepatitis B virus infection is associated with the presence of cell surface receptor sites that efficiently bind viral particles. J. Virol. *69*, 4814–4822.

OMTN, Volume *8*

# Supplemental Information

# Nucleic Acid Polymers with Accelerated Plasma and Tissue Clearance for Chronic Hepatitis B Therapy

**Ingo Roehl, Stephan Seiffert, Celia Brikh, Jonathan Quinet, Catherine Jamard, Nadine Dorfler, Jennifer A. Lockridge, Lucyna Cova, and Andrew Vaillant**

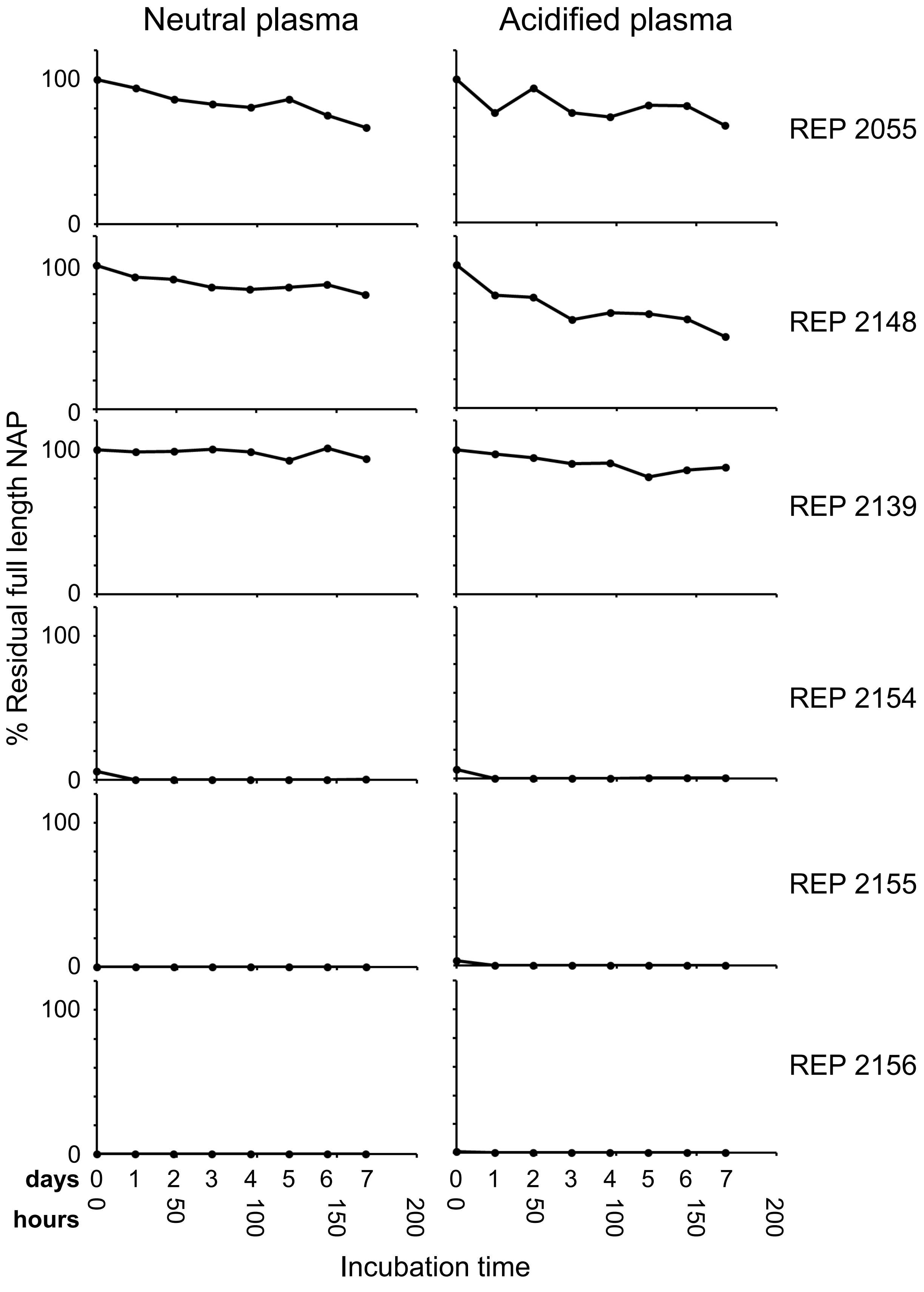


Figure S1. Stability of various NAPs in neutral and acidified human plasma over 7 days (part 1 of 3).

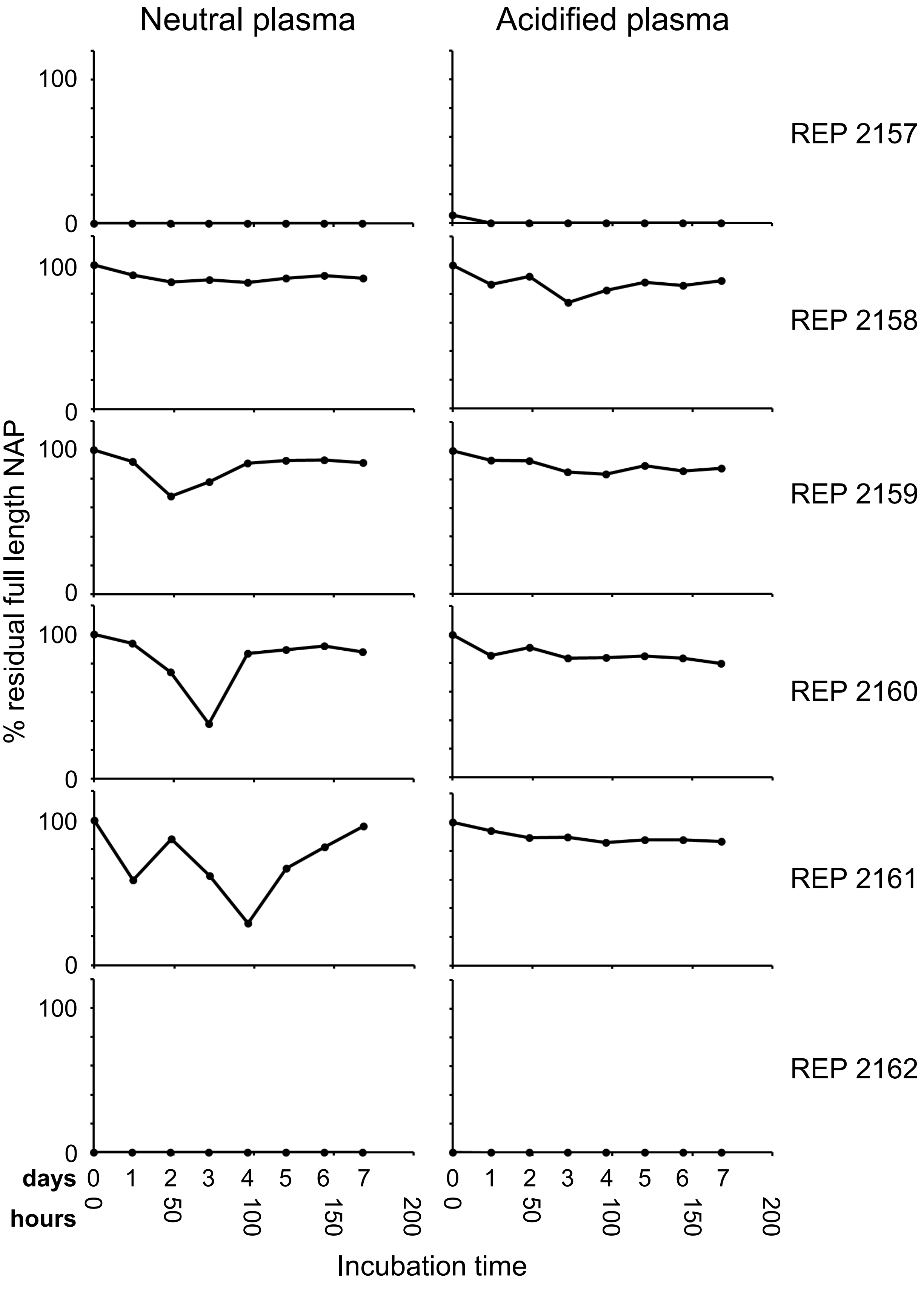


Figure S2. Stability of various NAPs in neutral and acidified human plasma over 7 days (part 2 of 3).

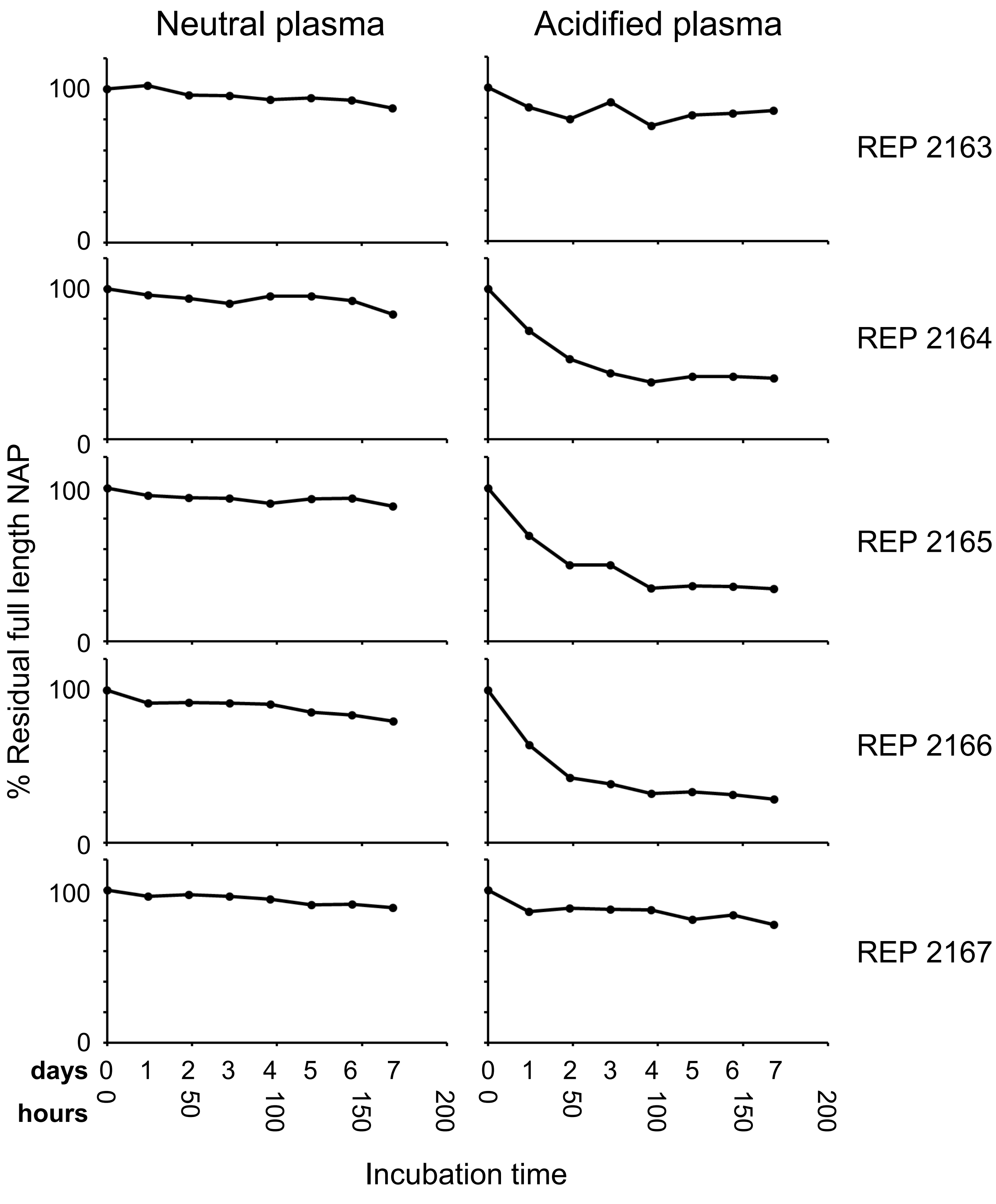


Figure S3. Stability of various NAPs in neutral and acidified human plasma over 7 days (part 3 of 3).

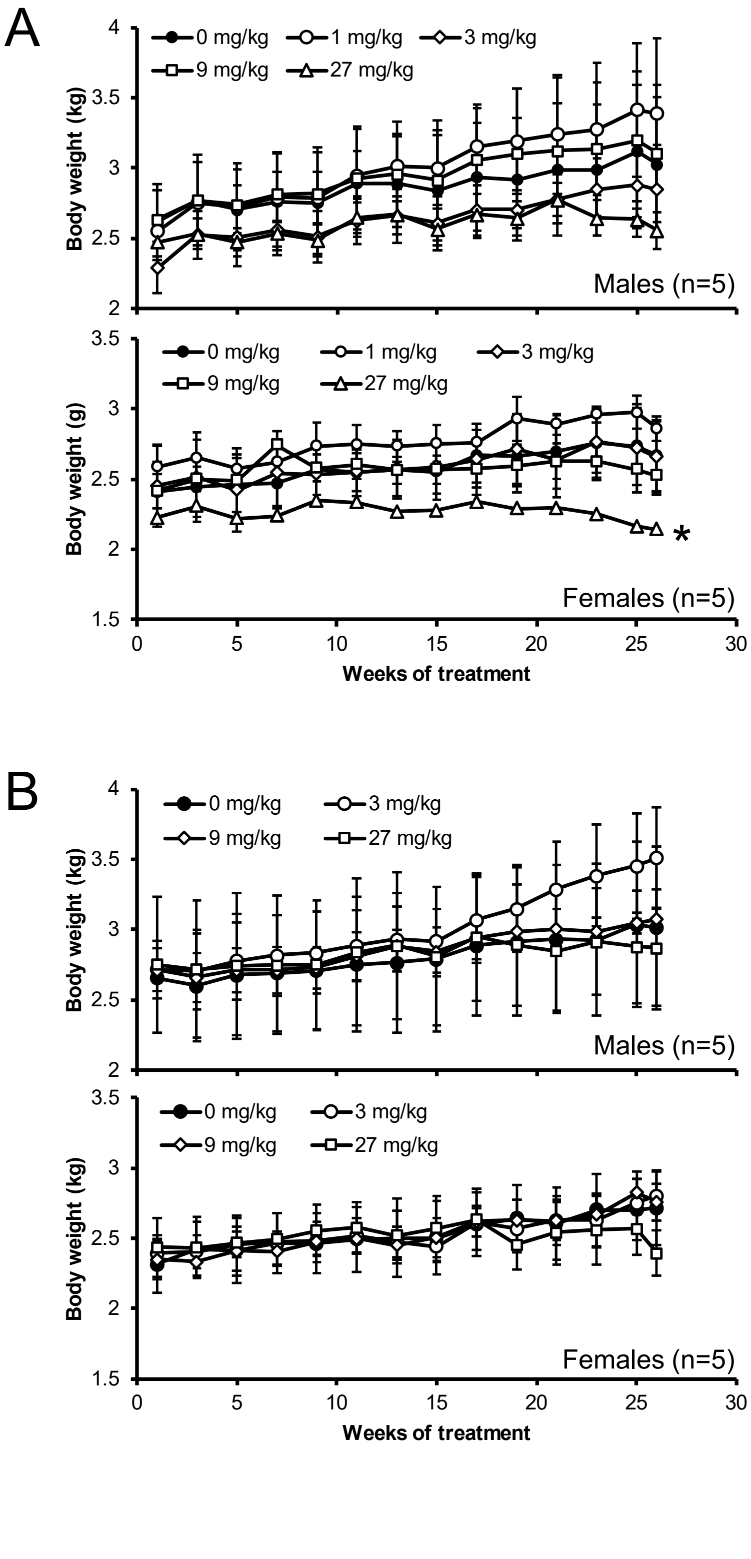


Figure S4. Body weight changes in cynomolgus monkeys over 26 weeks in the presence of various doses of REP 2139 (A) and REP 2165 (B) administered weekly by intravenous infusion. Mean and standard deviation are plotted separately for and females. Weight trends significantly different ($p < 0.05$, as determined by one-way ANOVA) from the 0 mg/kg control are indicated with an asterisk.

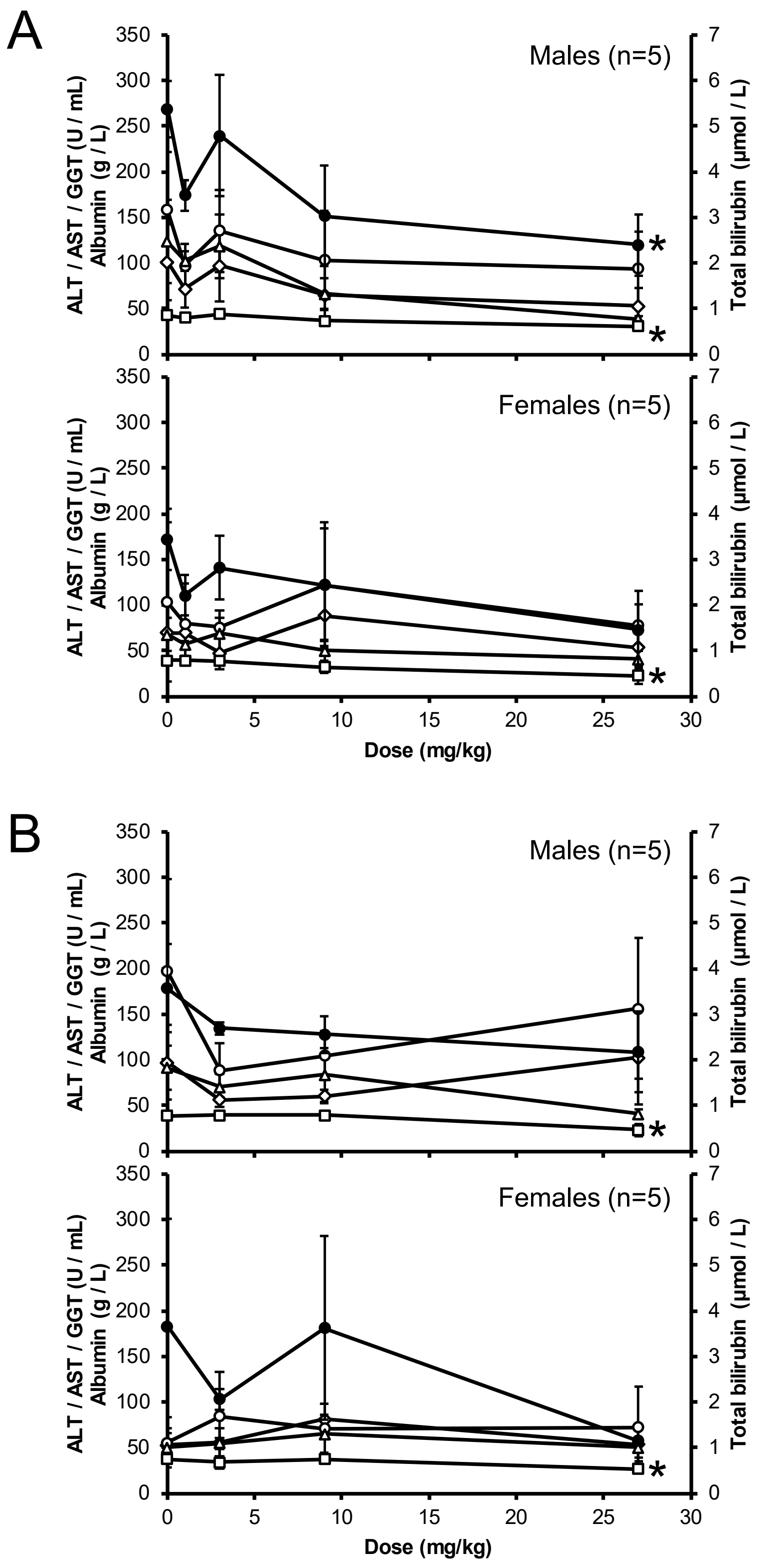


Figure S5. Liver function changes in cynomolgus monkeys at the end of 26 weeks of various doses of REP 2139 (A) and REP 2165 (B) administered weekly by intravenous infusion. Mean and standard deviation of ALT (diamonds), AST (open circles), GGT (triangles), albumin (squares) and total bilirubin (closed circles) are plotted separately for males and females. Trends significantly different ($p < 0.05$, as determined by one-way ANOVA) from the 0 mg/kg control are indicated with an asterisk.

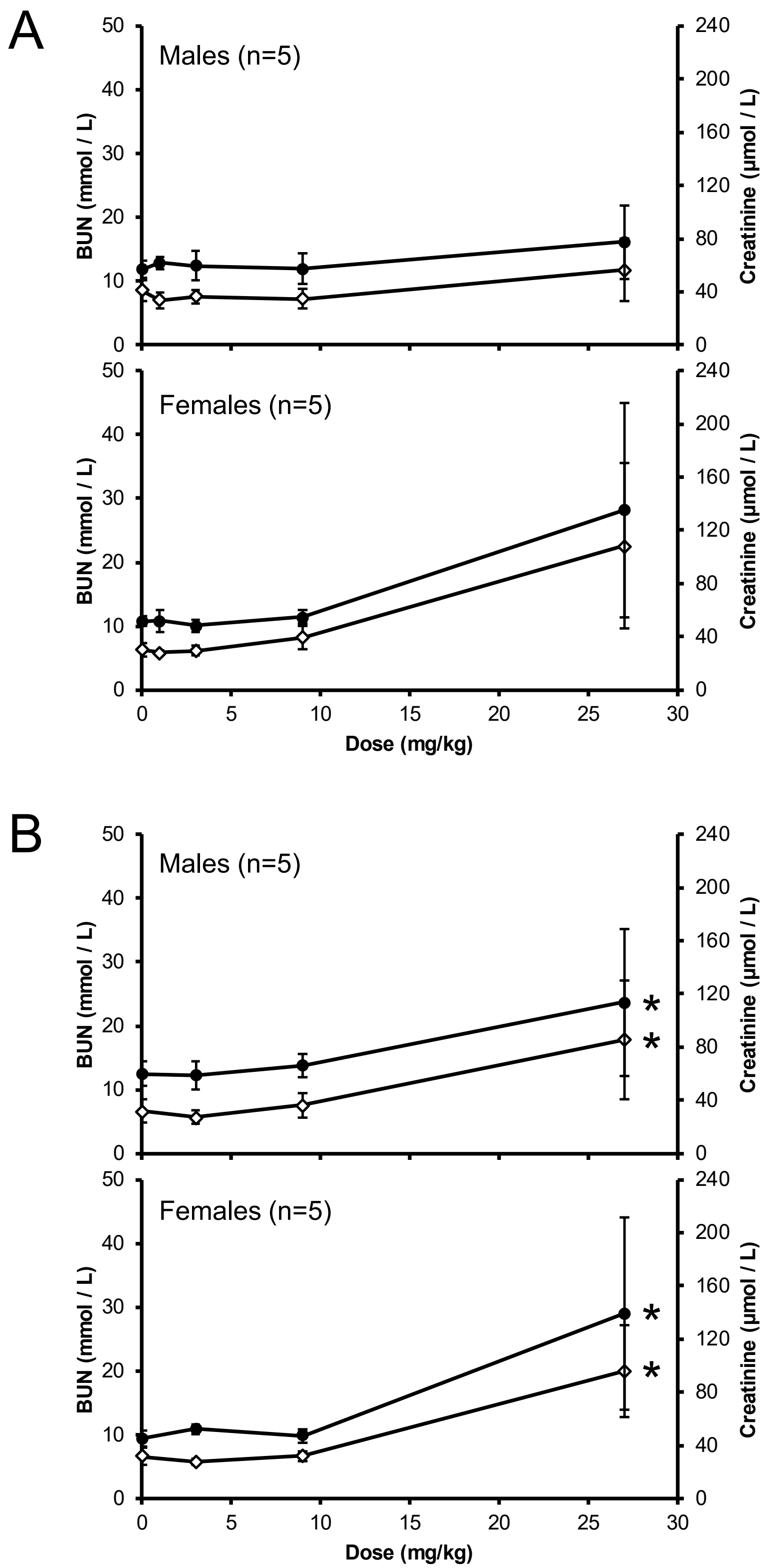


Figure S6. Kidney function changes in cynomolgus monkeys at the end of 26 weeks of various doses of REP 2139 (A) and REP 2165 (B) administered weekly by intravenous infusion. Mean and standard deviation of BUN (diamonds) and creatinine (closed circles) are plotted separately for males and females. Trends significantly different ($p < 0.05$, as determined by one-way ANOVA) from the 0 mg/kg control are indicated with an asterisk. BUN and creatinine increases in females at 27 mg/kg (whether significant or not) were considered clinically relevant.

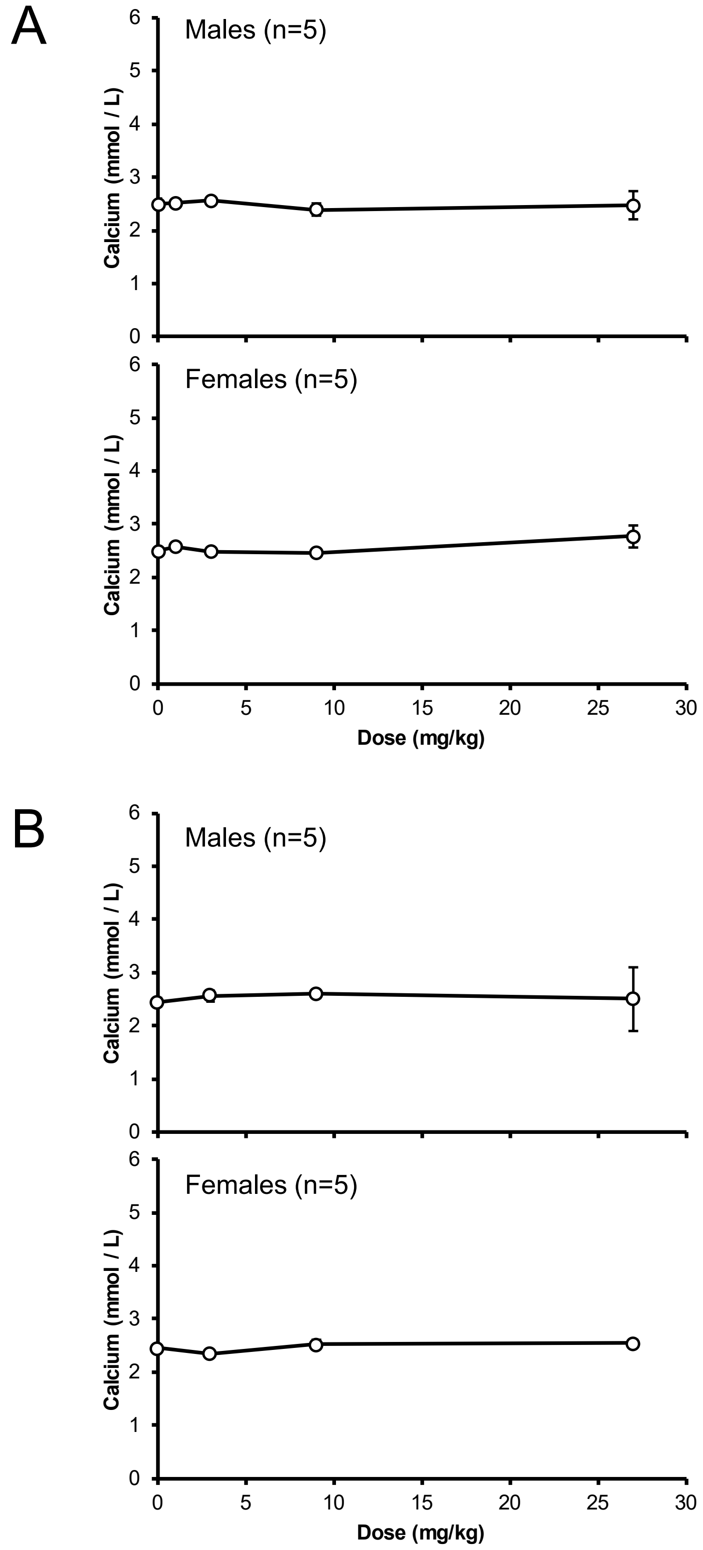


Figure S7. Serum calcium changes in cynomolgus monkeys at the end of 26 weeks of various doses of REP 2139 (A) and REP 2165 (B) administered weekly by intravenous infusion. Mean and standard deviation are plotted separately for males and females.

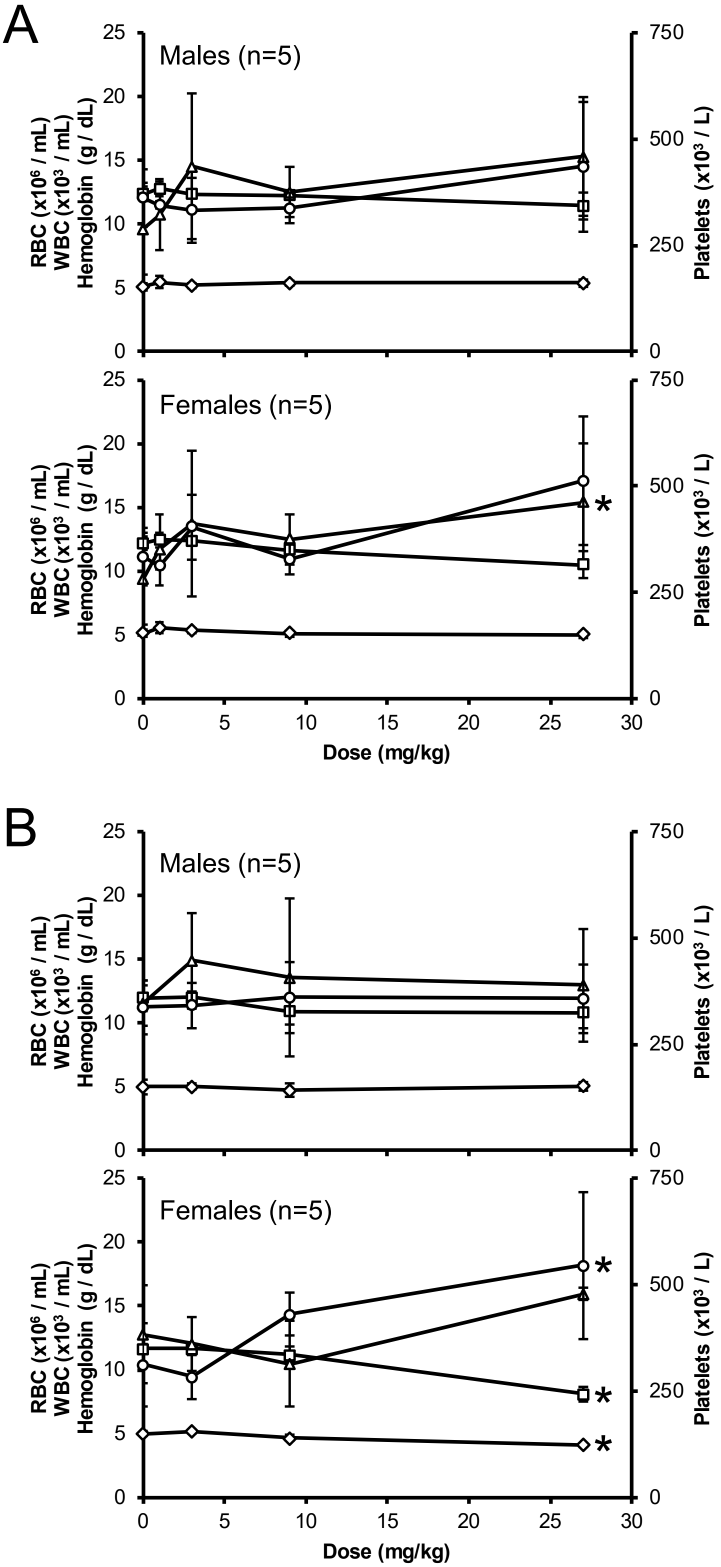


Figure S8. Hematological changes in cynomolgus monkeys at the end of 26 weeks of various doses of REP 2139 (A) and REP 2165 (B) administered weekly by intravenous infusion. Mean and standard deviation of RBC (diamonds), WBC (triangles), hemoglobin (squares), and platelets (circles) are plotted separately for males and females. Trends significantly different ($p < 0.05$, as determined by one-way ANOVA) from the 0 mg/kg control are indicated with an asterisk. Alterations in RBC and platelets at the 27mg/kg dose were not considered adverse.
